# Dissection for Floral Micromorphology and Plastid Genome of Valuable Medicinal Borages Arnebia and Lithospermum (Boraginaceae)

**DOI:** 10.3389/fpls.2020.606463

**Published:** 2020-12-04

**Authors:** Inkyu Park, Sungyu Yang, Jun-Ho Song, Byeong Cheol Moon

**Affiliations:** Herbal Medicine Resources Research Center, Korea Institute of Oriental Medicine, Naju, South Korea

**Keywords:** Boraginaceae, micromorphology, plastid genome, phylogenetic analysis, molecular marker

## Abstract

The genera *Arnebia* and *Lithospermum* (Lithospermeae-Boraginaceae) comprise 25–30 and 50–60 species, respectively. Some of them are economically valuable, as their roots frequently contain a purple-red dye used in the cosmetic industry. Furthermore, dried roots of *Arnebia euchroma*, *A. guttata*, and *Lithospermum erythrorhizon*, which have been designated Lithospermi Radix, are used as traditional Korean herbal medicine. This study is the first report on the floral micromorphology and complete chloroplast (cp) genome sequences of *A. guttata* (including *A. tibetana*), *A. euchroma*, and *L. erythrorhizon*. We reveal great diversity in floral epidermal cell patterns, gynoecium, and structure of trichomes. The cp genomes were 149,361–150,465 bp in length, with conserved quadripartite structures. In total, 112 genes were identified, including 78 protein-coding regions, 30 tRNA genes, and four rRNA genes. Gene order, content, and orientation were highly conserved and were consistent with the general structure of angiosperm cp genomes. Comparison of the four cp genomes revealed locally divergent regions, mainly within intergenic spacer regions (*atpH-atpI*, *petN-psbM*, *rbcL-psaI*, *ycf4-cemA*, *ndhF-rpl32*, and *ndhC-trnV-UAC*). To facilitate species identification, we developed molecular markers *psaA*- *ycf3* (PSY), *trnI-CAU- ycf2* (TCY), and *ndhC*-*trnV-UAC* (NCTV) based on divergence hotspots. High-resolution phylogenetic analysis revealed clear clustering and a close relationship of *Arnebia* to its *Lithospermum* sister group, which was supported by strong bootstrap values and posterior probabilities. Overall, gynoecium characteristics and genetic distance of cp genomes suggest that *A. tibetana*, might be recognized as an independent species rather than a synonym of *A. guttata*. The present morphological and cp genomic results provide useful information for future studies, such as taxonomic, phylogenetic, and evolutionary analysis of Boraginaceae.

## Introduction

*Arnebia* Forssk., an economically and medicinally important plant group of the Boraginaceae Juss. (tribe Lithospermeae Dumort.) comprises 25–30 species, distributed across Southwest and Central Asia, the Himalayas, Northeast Africa, and (Southeast) Mediterranean (Zhu et al., 1995; Mabberley, 2008; Ambrish and Srivastava, 2014; Coppi et al., 2015). Plants in the *Arnebia* are distinguishable from other Boraginaceae based on an actinomorphic corolla devoid of appendages, di- or tetra-stigmatic flowers, a style divided in two or four parts, a fruiting calyx often hardened and enclosing the nutlets, and nutlets without stipe (Forsskål, 1775; Zhu et al., 1995; Coppi et al., 2015). The roots of certain *Arnebia* taxa, which frequently contain a purple-red dye, have been used as a colorant in food and cosmetic preparations. Moreover, anti-microbial, anti-inflammatory, anti-oxidant, wound healing, anti-tumor, cardiotonic, and antineoplastic properties have been reported for *A. benthamii* (Wall. ex G. Don) I.M. Johnst. (Katoch et al., 2016), *A. densiflora* Ledeb. (Akkol et al., 2009; Kosger et al., 2009), *A. euchroma* (Royle ex Benth.) I.M. Johnst. (Damianakos et al., 2012; Bo et al., 2019), *A. hispidissima* (Lehm.) DC. (Jain et al., 2000; Shukla et al., 2001), and *A. nobilis* Rech.f. (Khatoon et al., 2003; Garg et al., 2014). The roots of *Lithospermum* L., another genus in the tribe Lithospermeae, also contain a red dye and have been reported to possess various phytomedical activities (Ishida and Sakaguchi, 2007). The dried roots of *A. euchroma*, *A. guttata* Bunge, and *Lithospermum erythrorhizon* Siebold & Zucc. are used as an important herbal remedy, known as Lithospermi Radix or Arnebiae Radix, which is designated as a medicine in the Korean and Chinese Herbal Pharmacopeia (Song J. H. et al., 2019; Korea Institute of Oriental Medicine [KIOM], 2020). Because their morphological, micromorphological, and molecular characteristics have not been compared and described in detail yet, the different species of medicinal borages have been used as a single remedy. Thus, there is a need to conduct comprehensive comparisons of their biological properties. Moreover, revealing the taxonomic identity of *A. tibetana* Kurz is essential to determine its medicinal properties. The taxonomic status of this species has been controversial. Several authors place *A. tibetana* as a synonym of *A. guttata* (Kazmi, 1970; Ambrish and Srivastava, 2014; Urgamal et al., 2014), whereas others consider that the biennial habitat and absence of blackish-purple spots on corolla lobes of *A. tibetana* support its classification as a separate species, independent from *A. guttata* (Zhu et al., 1995; Lazkov and Sultanova, 2014).

Floral micromorphological characteristics observed using scanning electron microscopy (SEM) have proven to be useful for taxonomic delimitation and phylogenetic inference of various groups, including Adoxaceae (Konarska, 2017), Crassulaceae (Giuliani et al., 2018), Nymphaeaceae (Coiro and Barone Lumaga, 2018), Lentibulariaceae (Płachno et al., 2019), and Rosaceae (Song et al., 2020). Notably, taxonomic and systematic relevance of floral micromorphology, including that of the corolla, calyx, style, and stigma, has been confirmed within Boraginaceae (Bigazzi et al., 1999; Bacchetta et al., 2000; Bigazzi and Selvi, 2000; Akçin, 2009).

Chloroplasts are key organelles in plants, where they mediate photosynthesis, carbon fixation, and biosynthesis of starch, fatty acids, amino acids, and pigments (Jansen and Ruhlman, 2012; Daniell et al., 2016). Chloroplast (cp) genomes of angiosperms reach 72 to 217 kb in size (Sugiura, 1992, 1995) and exhibit a quadripartite structure consisting of a large single-copy (LSC) and small single-copy (SSC) of a large inverted repeat (IR) region. Most angiosperm cp genomes contain approximately 80 protein-coding genes, four ribosomal RNA (rRNA) genes, and 30 transfer RNA (tRNA) genes (Wicke et al., 2011). The angiosperm cp genome structure and gene content are highly conserved due to lack of recombination, maternal inheritance, and elevated copy numbers (Wolfe et al., 1987; Delannoy et al., 2011). Hence, cp genomes have facilitated species classification, construction of high-resolution phylogenetic trees, and identification of evolutionary relationships in Plantae (Jansen et al., 2007; Parks et al., 2009). Furthermore, comparative studies of cp genomes provide useful information on gene rearrangements, including expansion or contraction of IR regions, as well as gene content, gene duplication or loss, genetic variance caused by insertion/deletion (indel), single sequence repeats (SSR), and single nucleotide polymorphism. These genetic markers can be used for species identification and classification, population genetics, and evolutionary studies (Hamilton et al., 2003; Chen et al., 2013). The remarkable reproducibility of methods based on these markers has facilitated the authentication and quality control of herbal medicine (Park et al., 2018a,b). Several studies have compared cp genomes to develop indel markers aimed at identifying herbal medicinal plants (Kim et al., 2015; Park et al., 2017, 2018b) and promoting their conservation.

The family Boraginaceae comprises 90 genera and 1,600 to 1,700 species according to the Angiosperm Phylogeny Group (APG) IV classification system (Cohen, 2014). Previous studies based on internal transcribed spacer (ITS) regions and a few cp loci have clarified the evolutionary relationships of Boraginaceae. Cohen (2014) classified 224 species of Boraginaceae based on their morphological characteristics, whereas Chacón et al. (2016) reclassified the monophyletic relationship between previously recognized clades and the position of various unplaced genera using 170 Boraginaceae species. However, lack of genomic resources has prevented the resolution of existing controversies regarding the *Arnebia* genus (Coppi et al., 2015). At present, the cp genome has been fully sequenced solely in *Borago officinalis* (NC_046796.1), *Onosma fuyunensis* (NC_049569.1), and *Tiquilia plicata* (MG573056). Only additional cp genome data will reveal the phylogenetic relationship between *Arnebia* and other genera in the Boraginaceae family, as well as correct species classification.

Here, we conducted a microscopic analysis of the floral organs of *A. guttata*, *A. tibetana*, *A. euchroma*, and *L. erythrorhizon*, and sequenced the cp genomes of these species. Moreover, we employed this information to verify the taxonomic and phylogenetic identity of *A. guttata* and *A. tibetana*.

## Materials and Methods

### Plant Material

Plant material used in this study was collected from natural populations in Mongolia, Kyrgyzstan, and Korea during a three-year survey of the vascular flora of Central Asia (2018–2020). Protologues and other relevant taxonomic literature were searched and used for accurate identification of all studied species (Royle, 1839; Bunge, 1840; Kurz, 1874; Johnston, 1924; Fang and Staples, 1995; Song J. H. et al., 2019; Kim, 2019). To confirm the consistency of all studied characteristics, at least two populations were compared for each species: *A. guttata* (two individuals), *A. tibetana* (10 individuals), *A. euchroma* (ten individuals), and *L. erythrorhizon* (10 individuals).

The samples were assigned identification numbers, and voucher specimens were deposited in the Korean Herbarium of Standard Herbal Resources (Index Herbariorum code: KIOM) at the Korea Institute of Oriental Medicine, Naju, South Korea. Moreover, the same collections were also deposited in both Institute for Biology of National Academy of Science (FRU) and the National University of Mongolia (UBU). Information about samples used for morphological, micromorphological, and cp genome sequence analyses is listed in Supplementary Table 1.

### Microscopic Analysis

For detailed floral micromorphological observations, fully mature reproductive organs were examined using a stereo microscope (SZX16; Olympus, Tokyo, Japan). For scanning electron microscopic observations, the dried samples from voucher specimens were rehydrated overnight in a wetting agent (Agepon: distilled water, 1:200; Agfa Gevaert, Leverkusen, Germany). The floral samples were dehydrated through a graded ethanol series (50, 70, 90, 95, and 100% ethanol) at room temperature for 1 h at each ethanol concentration. The dehydrated material was immersed in liquid CO_2_ for critical-point drying (SPI-13200JE-AB; SPI Supplies, West Chester, PA, United States) and subsequent mounting on aluminum stubs using a double-sided adhesive conductive carbon disk (05073-BA; SPI Supplies). All samples were coated with gold using an ion-sputtering device (208HR; Cressington Scientific Instruments Ltd., Watford, United Kingdom) and were observed using a low-voltage field emission scanning electron microscope (JSM-7600F; JEOL, Tokyo, Japan) at an accelerating voltage of 5–10 kV and working distance of 8–10 mm.

### Genome Sequencing and Assembly

Total DNA of the four tested species was extracted using the modified CTAB method (Allen et al., 2006). Four libraries were prepared from total genomic DNA using the TruSeq DNA Nano kit following the manufacturer’s protocols and generated using the NextSeq500 platform (Illumina, San Diego, CA, United States), generating 1.6–2 Gb of paired-end (2 × 150 bp) reads. The generated reads were trimmed and their quality was checked using CLC quality trim implemented in the CLC Assembly Cell software (ver 4.2.1; CLC Inc., Aarhus, Denmark). Trimmed paired-end reads (Phred score ≥ 20) were assembled using the CLC genome assembler (ver. 4.2.1; CLC Inc.) with default parameters. SOAP *de novo* gap closer was used to fill gaps based on alignment of paired-end reads (Luo et al., 2012). Contigs were aligned against NCBI nrDB to detect cp contigs and were retrieved from total contigs using Nucmer (Delcher et al., 2003). Aligned contigs were ordered using the cp genome sequences of *Abeliophyllum distichum* (MK616470.1), *Schrebera orientalis* (NC_042266.1), and *Forestiera ligustrina* (MH817903.1) as references. The complete cp genomes were validated by PCR-based sequencing using sequence-specific primers (Supplementary Table 2). PCR products of the four junctions (LSC/IRa, IRa/SSC, SSC/IRb, and IRb/LSC) were compared to complete cp genome sequences (Supplementary Table 3). Finally, total trimmed paired-end reads were mapped onto complete genome sequences using BWA ver. 0.7.25 (Li and Durbin, 2010; Supplementary Figure 1).

### Genome Annotation and Comparative Analysis

Gene annotation of *A. guttata*, *A. tibetana*, *A. euchroma*, and *L. erythrorhizon* cp genomes was performed using GeSeq (Tillich et al., 2017), and annotation results were concatenated using an in-house script pipeline. Protein-coding sequences (CDS) were manually curated and confirmed using Artemis (Carver et al., 2008), and checked against the NCBI protein database. The tRNAs were confirmed with tRNAscan-SE 1.21 (Lowe and Eddy, 1997). IR region sequences were confirmed using IR finder and RepEx (Gurusaran et al., 2013). Circular maps of the three *Arnebia* and one *Lithospermum* cp genomes were obtained using OGDRAW (Greiner et al., 2019). GC content and relative synonymous codon usage (RSCU) of the four cp genomes were analyzed using MEGA7 software (Kumar et al., 2016). Codon usage distribution of six Boraginaceae cp genomes was visualized using the Heatmapper program, with average linkage of clustering and Euclidean distance measurement methods (Babicki et al., 2016). RSCU < 1.00 indicated a codon that was used less frequently than expected; whereas RSCU > 1.00 indicated a codon that was used more frequently than expected. MAUVE V2.3.1 was used to identify local collinear blocks of six Boraginaceae species (*Arnebia guttata*, *A. tibetana*, *A. euchroma*, *Lithospermum erythrorhizon*, *Borago officinalis*, and *Tiquilia plicata*; Darling et al., 2010). The mVISTA program in Shuffle-LAGAN mode was used to compare the cp genomes with *A. guttata* as reference (Frazer et al., 2004). DnaSP version 6 was used to calculate nucleotide variability (Pi) among cp genomes (Rozas et al., 2017). Substitution rates Ka and Ks were estimated with KaKs Calculator ver. 2.0 (Wang et al., 2010).

### Repeat Analysis

We used REPuter to find forward and reverse repeats with minimal length of 20 bp, identity of 90%, and a Hamming distance of 3 (Kurtz et al., 2001). SSRs were detected using MISA (Beier et al., 2017), with the minimum number of repeat parameters set to 10, 5, 4, 3, 3, and 3 for mono-, di-, tri-, tetra-, penta-, and hexanucleotides, respectively. Tandem repeats ≥20 bp were identified using Tandem repeats finder (Benson, 1999) with minimum alignment score of 50 and maximum period size of 500; the identity of repeats was set to ≥90%.

### Development of Molecular Markers and Validation for *Arnebia* and *Lithospermum*

Primers for indel markers were designed using NCBI Primer-BLAST. Specificity of indel markers was confirmed by PCR with 20 ng of genomic DNA extracted from eight samples of the four species. Reactions were carried out in a 20-μL PCR mixture with 10 pmol of PSY, TCY, and NCTV primers using a ProFlex PCR system (Applied Biosystems, Waltham, MA, United States) with the following amplification parameters: initial denaturation at 95^°^C for 2 min; 35 cycles at 95^°^C for 50 s, 60^°^C for 50 s, and 72^°^C for 50 s; final extension at 72^°^C for 5 min. PCR products were separated on a 2% agarose gel for 40 min at 150 V. The germplasms of the three Arnebia and one Lithospermum species are listed in Supplementary Table 4. PSY, TCY, and NCTV primer sequences are listed in Supplementary Table 5.

### Phylogenetic Analysis

A total of eight cp genomes, namely seven from Boraginaceae, one from Oleaceae, were used for phylogenetic analysis, along with *Forsythia suspensa* (NC_036367) as outgroup. Of these, 4 chloroplast genome sequences were downloaded from the NCBI GenBank (Supplementary Table 6). We used two matrices, one composed of 72 conserved protein-coding genes (CDS) and one of the whole chloroplast genomes. Using MAFFT ver. 7.388 (Katoh and Standley, 2013) two data set were aligned, respectively. Each aligned gene was extracted using Geneious program^1^, and then the genes were arranged alphabetically. The concatenated gene dataset was created using Geneious program in CDS data set. The alignments data sets were filtered to remove ambiguously aligned regions using Gblocks ver. 5 (Castresana, 2000). The best-fitting model of nucleotide substitutions was determined using Akaike information criterion in jModelTest V2.1.10 (Darriba et al., 2012; Supplementary Table 7) and the GTR+I+G model was chosen for Maximum likelihood (ML) analysis and GTR+G model was selected for Bayesian Inference (BI) analysis. ML analysis was performed using RaxML v 8.0.5 (Stamatakis, 2014) with 1,000 bootstrap replicates. BI analysis was carried out using MrBayes 3.2.2 (Ronquist et al., 2012), with two independent runs of four simultaneous chains that were run for 5,000,000 generations using Markov chain Monte Carlo algorithm. Trees were sampled every 5,000 generations, with the first 25% discarded as burn-in. Trees were determined from 50% majority-rule consensus to estimate posterior probabilities. The reconstructed trees were visualized using Figtree V.1.4.2 (Rambaut, 2014).

## Results

### Floral Characteristics of *Arnebia* and *Lithospermum*

**FIGURE 1:**
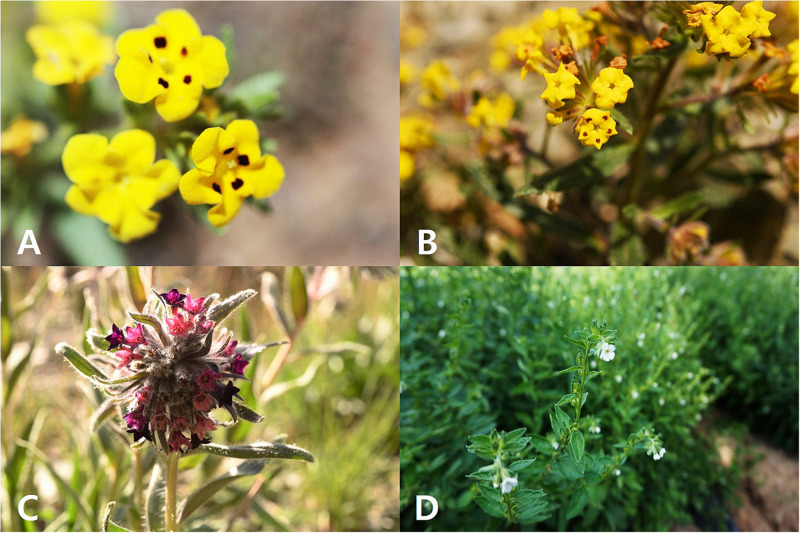
Flowers of *Arnebia* and *Lithospermum* species. **(A)** *Arnebia guttata*, **(B)** *Arnebia tibetana*, **(C)** *Arnebia euchroma*, and **(D)** *Lithospermum erythrorhizon.*

**FIGURE 2:**
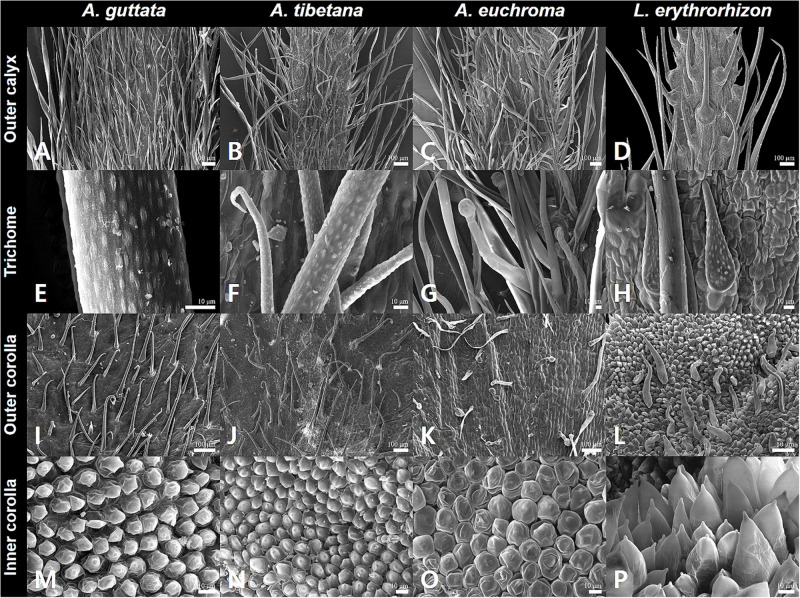
Calyx and corolla micromorphological characteristics of *Arnebia* and *Lithospermum* species. **(A,E,I,M)** *Arnebia guttata*, **(B,F,J,N)** *Arnebia tibetana*, **(C,G,K,O)** *Arnebia euchroma*, and **(D,H,L,P)** *Lithospermum erythrorhizon.*

**FIGURE 3:**
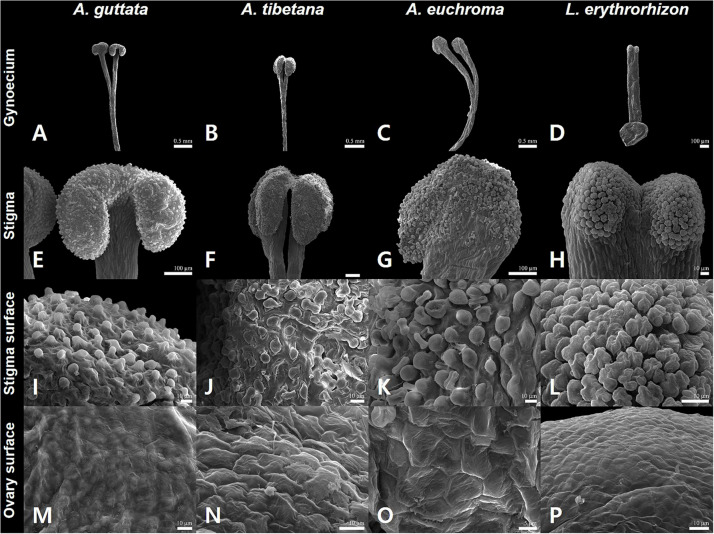
Gynoecium characteristics of *Arnebia* and *Lithospermum* species. **(A,E,I,M)** *Arnebia guttata*, **(B,F,J,N)** *Arnebia tibetana*, **(C,G,K,O)** *Arnebia euchroma*, and **(D,H,L,P)** *Lithospermum erythrorhizon.*

Floral external and epidermal micromorphological characteristics (calyx, corolla, and gynoecium surfaces) are summarized in Table 1, while representative photo- and micrographs are shown in Figures 1–3. Although limited variation was observed between individuals of the same species, the four species could be clearly distinguished by their floral characteristics.

**TABLE 1:** Floral morphological and micromorphological characteristics of *Arnebia* and *Lithospermum* species.

**Species**	***A. guttata***	***A. tibetana***	***A. euchroma***	***L. erythrorhizon***
Inflorescences	Cymes	Cymes	Cymes	Scorpioid cymes
Flower color	Yellow	Yellow	Purple	White
Calyx lobation	5-lobed	5-lobed	5-lobed	5-parted
Calyx lobe shape	Linear	Linear	Linear	Linear
Outer calyx surface	vTR	vTR	sGS, sTR	vTR, sHI
Corolla shape	Tubular-campanulate	Tubular-campanulate	Tubular-campanulate	Tubular-campanulate
Outer corolla cell	Tabular rugose	Tabular rugose	Tabular rugose	Papillose knobby
Outer corolla surface	vTR	vTR	sGS, sTR	vTR
Inner corolla cell	Papillose conical	Papillose conical	Papillose conical	Papillose knobby
Inner corolla surface	sGS	sGS	Gla	vTR, sGS
Style shape	Filiform	Filiform	Filiform	Linear
Style lobation	2-cleft	2-fid	2-cleft	Indistinct
Stigma number	2	2	2	2
Stigma shape	Oblate	Oblong	Globose	Globose
Stigma surface	Tuberculate	Tuberculate	Tuberculate	Asterisk- tuberculate
Ovary surface	Colliculate	Colliculate	Colliculate	Colliculate

*Trichome shape: Gla, glabrous; sGS, smooth glandular trichome with stalk; sTR, smooth non-glandular trichome; and vTR, verrucate non-glandular trichome.*

All studied borages presented cyme inflorescences: *L. erythrorhizon* had scorpioid cymes with paired flowers, *A. guttata* and *A. tibetana* displayed crowded cymes with many flowers, and *A. euchroma* exhibited terminal cymes with numerous flowers. The corolla was yellow with or without dark spots on corolla lobes in *A. guttata* and *A. tibetana* (Figures 1A,B), dark purple to purple in *A. euchroma* (Figure 1C), and white in *L. erythrorhizon* (Figure 1D). The calyx of the three *Arnebia* species was 5-lobed; whereas that of *L. erythrorhizon* was 5-parted and more deeply lobed than in *Arnebia* specimens.

The outer calyx of *A. guttata* and *A. tibetana* was densely covered with non-glandular trichomes (Figures 2A,B); whereas that of *A. euchroma* was densely covered with both non-glandular trichomes and glandular trichomes with stalk (Figure 2C). Hirsute, thick, and long non-glandular trichomes were found in *L. erythrorhizon* (Figure 2D). Non-glandular trichome surfaces were generally verrucate (Figures 2E,F); whereas glandular trichomes were always smooth (Figure 2G). Only in *L. erythrorhizon*, non-glandular trichomes were both verrucate and smooth (Figure 2H). The shape of the corolla was tubular-campanulate in all species; however, cellular and trichome distribution patterns were quite different. All *Arnebia* species presented a tabular rugose cellular pattern on the outer corolla (Figures 2I–K); whereas *L. erythrorhizon* exhibited a papillose knobby pattern (Figure 2L). The verrucate, non-glandular trichomes were sparsely to moderately pubescent on the outer corolla surface of *A. guttata*, *A. tibetana*, and *L. erythrorhizon*. Smooth glandular trichomes with stalk and smooth non-glandular trichomes were only found in *A. euchroma*. The inner corolla cells of all *Arnebia* species were papillose conical and 10.70 ± 2.69 (S.D.) μm tall (Figures 2M–O), whereas those of *L. erythrorhizon* were papillose knobby and 36.74 ± 6.22 (S.D.) μm tall (Figure 2P). *A. guttata* and *A. tibetana* had smooth glandular trichomes with stalk on the inner corolla surface, *A. euchroma* was glabrous, and *L. erythrorhizon* had both smooth glandular trichomes with stalk and verrucate non-glandular trichomes.

The gynoecium varied between species (Figure 3). All *Arnebia* species had a filiform style and two stigmas (Figures 3A–C); whereas *L. erythrorhizon* had an indistinct broad linear style with two stigmas (Figure 3D). Stylar lobation in *Arnebia* was of two types: 2-cleft (divided almost through the middle) in *A. guttata* (Figure 3A) and *A. euchroma* (Figure 3C), and 2-fid (divided through the top) in *A. tibetana* (Figure 3B). The stigma of *Arnebia* species presented different shapes: oblate in *A. guttata* (Figure 3E), oblong in *A. tibetana* (Figure 3F), and globose in *A. euchroma* (Figure 3G). In contrast, cellular surface patterns were of the same tuberculate type (Figures 3I–K). *L. erythrorhizon* had a globose stigma with asterisk-tuberculate cellular surface pattern (Figure 3L). All studied species shared a colliculate ovary surface (Figures 3M–P).

### Organization of *Arnebia* and *Lithospermum* Chloroplast Genome

**FIGURE 4:**
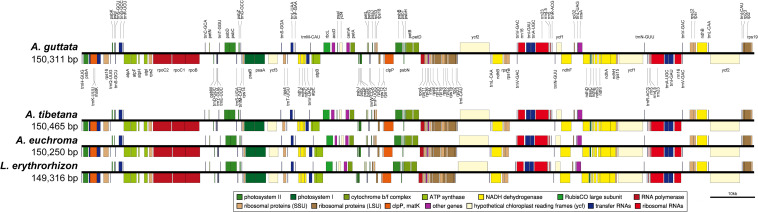
Linear gene map of chloroplast genomes from *Arnebia* and *Lithospermum* species. Genes are transcribed left to right. Genes above the line are positioned in the forward direction (left to right). Genes below the line are positioned in the reverse direction (right to left).

We sequenced four Boraginaceae accessions: *A. guttata*, *A. tibetana*, *A. euchroma*, and *L. erythrorhizon* at approximately 601X, 324X, 447X, and 433X coverage, respectively. This generated 1.6–2.0 Gb raw paired-end reads and 1.3–1.7 Gb trimmed reads (Supplementary Table 8). The complete circular cp genome was 149,316–150,465 bp and matched the quadripartite structure of cp genomes, consisting of a pair of IRs (17,144–17,311 bp) separated by LSC (80,411–81,348 bp) and SSC (25,856–25,979 bp) regions (Figure 4, Table 2, and Supplementary Figure 2). The complete cp genomes were all of high quality (Supplementary Figure 1). Overall GC content was approximately 37%, and slightly higher in the IR (43%) than LSC (35%) or SSC (31%) regions. The four cp genomes comprised 112 genes, namely 78 protein-coding, four rRNA, and 30 tRNA genes. They had 18 intron-containing genes, 15 of which had a single intron and two of which (*ycf3*, *clpP*) had three introns with duplicate genes (*ndhB*, *trnI-GAU*, and *trnA-UGC*) in the IR regions (Supplementary Table 10). Analysis of codon usage and anticodon recognition patterns revealed 24,529–25,929 codons, among which leucine, isoleucine, and serine were the most abundant (Supplementary Figure 3). To identify codon patterns, we analyzed codon distribution in six cp genomes using the hierarchical clustering method (Supplementary Figure 4). Red and green indicate weak (RSCU > 1) and strong (RSCU < 1) codon bias, respectively. Codons with an A or T in the third position had a strong codon bias. Most Boraginaceae displayed a similar pattern, with high RSCU values for GGT (Guanine), AGG (Arginine), and GGG (Guanine); only *B. officinalis* presented particularly low RSCU values.

**TABLE 2:** Features of *Arnebia* and *Lithospermum* chloroplast genomes.

**Species**	***A. guttata***	***A. tibetana***	***A. euchroma***	***L. erythrorhizon***
Accession number	MT975391	MT975392	MT975393	MT975394
Total cp genome size (bp)	150,311	150,465	150,250	149,316
Large single-copy (LSC) region (bp)	81,267	81,348	81,103	80,411
Inverted repeat (IR) region (bp)	17,144	17,159	17,311	17,193
Small single-copy (SSC) region (bp)	25,950	25,979	25,918	25,856
Total number of genes (unique)	112	112	112	112
Protein-coding genes (unique)	78	78	78	78
rRNA (unique)	4	4	4	4
tRNA (unique)	30	30	30	30
GC content (%)	37.7	37.7	37.6	37.6
LSC (%)	35.6	35.6	35.6	35.5
IR (%)	43.0	43.0	43.0	43.0
SSC (%)	31.4	31.3	31.1	31.2

**FIGURE 5:**
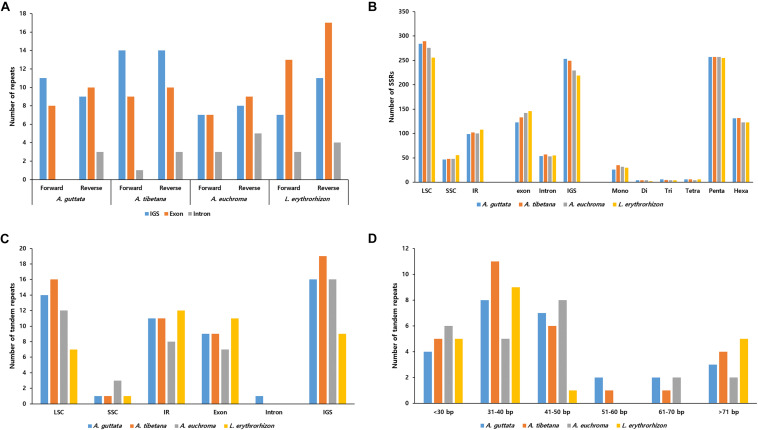
Distribution of repeat sequences in *Arnebia* and *Lithospermum* chloroplast genomes. **(A)** Number of forward and reverse repeats in genomic regions. **(B)** Distribution of SSRs in intergenic spacer (IGS), exon, and intron regions. **(C)** Distribution of tandem repeats in genomic regions. **(D)** Distribution of tandem repeats’ length.

REPuter-based analysis of repetitive sequences, such as forward and reverse repeats, yielded 39–55 repeats (Figure 5A). In the three *Arnebia* cp genomes, they were located in the intragenic spacer (IGS) region, while in *L. erythrorhizon* they were particularly abundant in exons. MISA software (Beier et al., 2017) identified a total of 420–439 SSRs (Figure 5B), mostly in the LSC and particularly within the IGS region. The pentanucleotide motif was the most abundant in all accessions, followed by hexanucleotide motif repeats (Figure 5C). Tandem repeats finder (Benson, 1999) located most tandem repeats in the LSC (Figure 5D). They were generally 31–50 bp long and only 2–5 tandem repeats >71 bp in length were identified in all four accessions.

### Comprehensive Comparative Analysis of *Arnebia* and *Lithospermum* Chloroplast Genomes

We compared IR contraction and expansion of the Boraginaceae accessions with those of *Forsythia suspensa* (Supplementary Figure 5). In spite of similar IR length, from 25,217 to 25,979 bp, differences in IR expansions and contractions were observed. The *rps19* gene of five Boraginaceae (*A*. *guttata*, *A*. *tibetana*, *A*. *euchroma*, *L. erythrorhizon*, and *B. officinalis*) was located entirely in the LSC region, but was expanded beyond that in *Tiquilia plicata*. The *ycf1* gene was located at the junction of IRb/SSC and SSC/IRa, with the border between IRa/SSC extending into *ycf1*. The IRb/SSC border was also located in the *ycf1* gene, but it expanded by the same length also into the *ψycf1* gene. Overall, IRb was found to be expanded in all cp genomes assessed. The *trnH* gene was located in the LSC region, 16–40 bp from the IRa/LSC boundary. The *rpl2* gene of *T. plicata* was duplicated in the IR regions flanking border junctions. Generally, *Arnebia* and *Lithospermum* appeared to be highly conserved.

**FIGURE 6:**
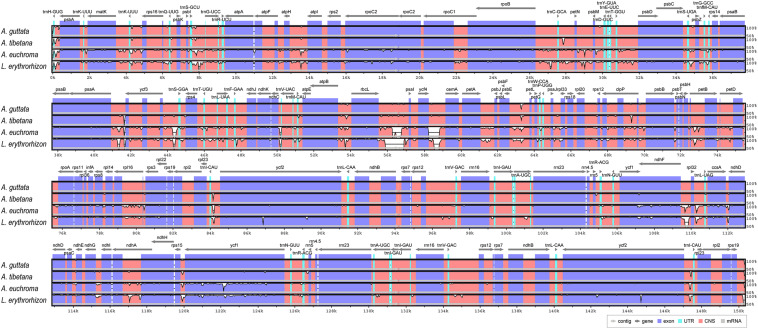
Comparison of *Arnebia* and *Lithospermum* chloroplast genomes using mVISTA. Complete cp genomes of five *Trichosanthes* were compared against *A. guttata* as reference. Blue block: conserved genes, sky-blue block: transfer RNA (tRNA) and ribosomal RNA (rRNA), and red block: conserved non-coding sequences (CNS). Regions with sequence variation among the five *Trichosanthes* are denoted in white.

**FIGURE 7:**
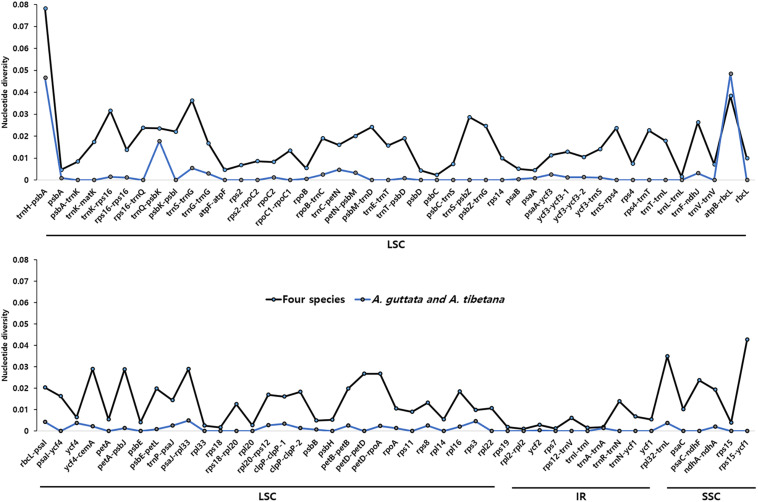
Comparison of nucleotide diversity (Pi) values among *Arnebia* and *Lithospermum* species. The dotted line indicates the average Pi value in *Arnebia* and *Lithospermum* chloroplast genomes and excludes regions with Pi = 0.

We analyzed sequence identity using MAUVE and mVISTA programs, with *A. guttata* as reference. Overall, Boraginaceae cp genome structure was well conserved (Figure 6 and Supplementary Figure 6), with genic regions being more conserved than IGS regions. Most genic regions were highly convergent; the only divergent regions were detected in *matK*, *rpoC2*, *rpoC1*, *ycf1*, *ycf2*, and *ndhF* genes. The highest divergence was observed in IGS regions, such as *atpH-atpI*, *petN-psbM*, *rbcL-psaI*, *ycf4-cemA*, *ndhF-rpl32*, and *ndhC-trnV-UAC*. The cp genomes of *A. guttata* and *A. tibetana* were particularly highly conserved. Mildly divergent regions were identified in *trnS-GCU-trnG-UCC*, *trnC-GCA-petN*, *psaA-ycf3*, *trnI-CAU-ycf2*, *psbE-petL*, *clpP*, and *rps15-ycf1* (Figure 6). Nucleotide diversity (Pi) for the cp genome of the four species was calculated as 0.015 using the DNA SP program. IR regions (Pi = 0.007) were more variable than LSC (Pi = 0.0046) or SSC (Pi = 0.019) regions. Single-copy regions presented higher Pi values than IR regions. In particular, *trnH-psbA* (Pi = 0.078) and *atpB-rbcL* (Pi = 0.038) appeared highly diverse (Figure 7). In spite of the morphological and cp genome similarity between *A. guttata* and *A. tibetana*, the *atpB-rbcL* region was more diverse (Pi = 0.048) than the average for the four cp genomes.

Using the non-synonymous substitution to synonymous substitution (Ka/Ks) ratio, a total of 77 genes were calculated to identify selective pressure for the three *Arnebia* species, *L. erythrorhizon*, and *B. officinalis*, with *T. plicata* as reference (Supplementary Figure 7). Most genes were conserved and exhibited a Ka/Ks ratio < 1. Only *rbcL* was positively selected in all five Boraginaceae cp genomes (Ka/Ks = 1.064–1.286).

### Development of Indel Marker Sets for Efficient Species Classification

**FIGURE 8:**
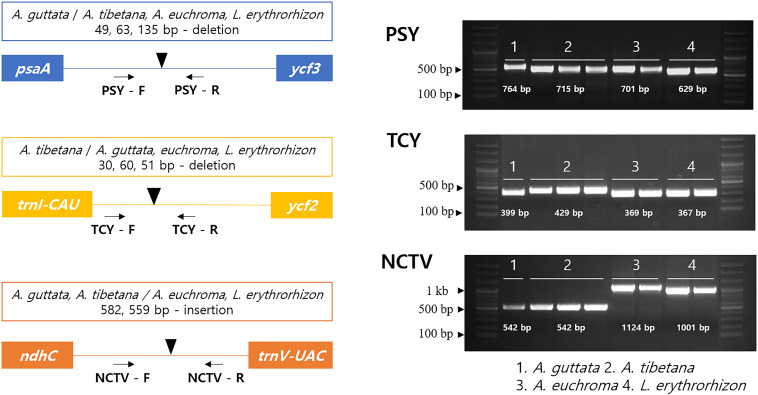
Schematic representation of indel markers for *Arnebia* and *Lithospermum* species. Primers for PSY (*psaA*- *ycf3*), TCY (*trnI-CAU-ycf2*), and NCTV (*ndhC-trnV-UAC*) were tested. Control samples were used in a marker test (Supplementary Table 4). 1: *A. guttata*; 2: *A. tibetana*; 3: *A. euchroma*; and 4: *L. erythrorhizon*.

Herbal medicinal plants represent important resources. To ensure efficient identification among the four tested species, we developed indel marker sets targeting three intergenic regions: *psaA*- *ycf3* (PSY), *trnI-CAU- ycf2* (TCY), and *ndhC*-*trnV-UAC* (NCTV; Figure 8). The PSY (*psaA*- *ycf3*) marker could discriminate clearly between the four species as it yielded fragments of 764 bp with *A. guttata*, 715 bp with *A. tibetana*, 701 bp with *A. euchroma*, and 629 bp with *L. erythrorhizon*. TCY (*trnI-CAU- ycf2*) was employed to distinguish *A. tibetana*, which yielded a fragment of 429 bp, from *A. guttata* (399 bp), *A. euchroma* (369 bp), and *L. erythrorhizon* (367 bp). Finally, NCTV (*ndhC*-*trnV-UAC*) could discriminate *A. euchroma* and *L. erythrorhizon* from *A. guttata* and *A. tibetana*.

### Phylogenic Relationships Among Boraginaceae

**FIGURE 9:**
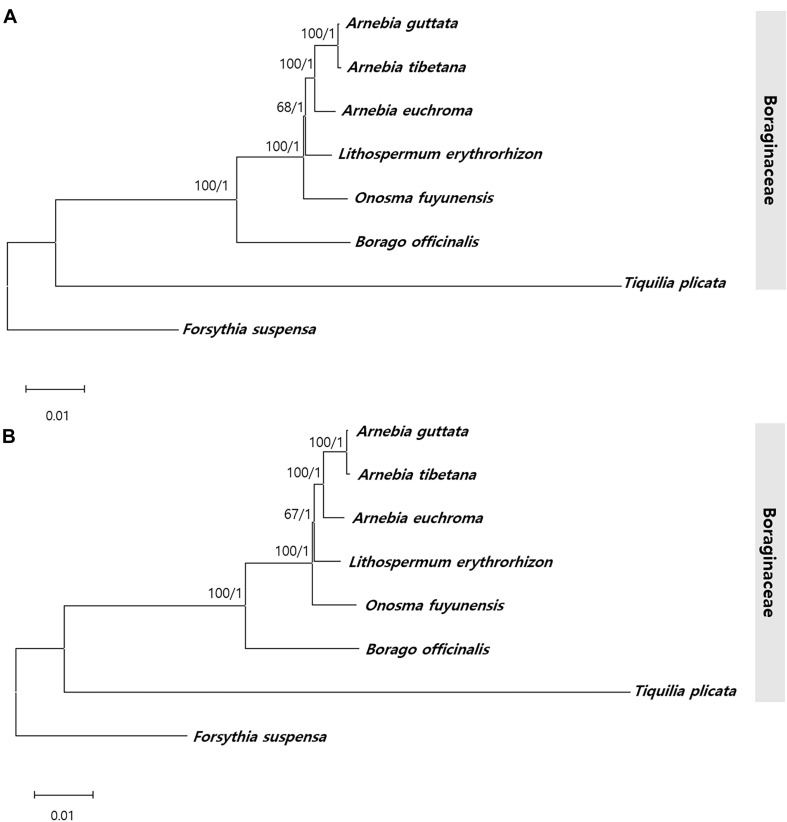
Phylogenetic tree from *Arnebia* and *Lithospermum* using maximum likelihood bootstraps and Bayesian posterior probabilities. **(A)** Whole chloroplast genome dataset topology, **(B)** 72 CDS dataset topology. Maximum likelihood topology is shown with bootstrap support values/Bayesian posterior probabilities given at each node.

To identify the phylogenic relationships between *Arnebia* and *Lithospermum* species within the Boraginaceae family, we aligned the whole chloroplast genomes and 71 conserved protein- CDS (65,155 bp) shared by the three *Arnebia* and one *Lithospermum* accessions examined in this study, with *F. suspensa* from the Oleaceae used as outgroup (Figure 9 and Supplementary Figure 8). ML and BI topologies were highly congruent for both the whole chloroplast genomes and CDS data. All phylogenetic trees except *Lithospermum* were strongly supported (ML = 100%, BI = 1.0). Seven species within the Boraginaceae clustered consistently with respect to the APG IV classification system. Overall, clustering revealed that *A. guttata* and *A. tibetana* shared the closest phylogenetic relationship, with *A. euchroma* as their sister group and *Lithospermum* clearly distinct from *Arnebia*.

## Discussion

### Floral Characteristics of *Arnebia* and *Lithospermum* Species

Although all studied species shared the same calyx lobe or corolla shape and ovary surface cell patterns, *Arnebia* and *Lithospermum* exhibited substantial diversity with respect to floral epidermis cell patterns, gynoecium, and structure of trichomes.

Based on floral morphology, only *L. erythrorhizon* had scorpioid cymes and white flowers with faucal appendages (also referred to as “faucal scale”) on the corolla tube, deeply lobed calyx, and indistinct linear-shaped style (Table 1). Faucal appendages, which appear in certain species, represent a useful diagnostic tool in Boraginaceae (Zhu et al., 1995; Cohen, 2011; Weigend et al., 2016). Previous studies have suggested that these appendages could be modified through development of specialized epidermal cells to better attract pollinators (Kelley and Wilken, 1993; Cohen, 2011). Accordingly, *L. erythrorhizon* with faucal appendages and *Arnebia* species without faucal appendages may attract different pollinators. In *Arnebia*, *A. euchroma* could be easily distinguished from other species based on morphological traits, including corolla color, plant height, root length, and leaf shape (Song J. H. et al., 2019).

The observations of members of the tribe Boragineae (genera *Gastrocotyle*, *Hormuzakia*, and *Phyllocara*) have revealed that the floral trichome characteristics, such as trichome surface pattern, density, length, and cell number, have taxonomic implications in the context of their generic classification (Bigazzi et al., 1999). Our floral micromorphological data also confirmed the usefulness of trichome types for the identification of medicinal borage species. The morphology and the distribution of trichomes in *A. guttata* were similar to those of *A. tibetana* (verrucate, non-glandular trichome on both the outer calyx and corolla). In contrast, smooth, hirsute non-glandular trichomes on the outer calyx were exclusive to *L. erythrorhizon*; whereas non-glandular and glandular trichomes with stalk on both the outer calyx and corolla were unique to *A. euchroma*. Hirsute, rigid trichomes form physical barriers against insects as part of a “greasy pole strategy” (Kerner, 1876; Harley, 1991; Eigenbrode, 2004; Jeffree, 2006). Floral glands, which contain glucose or phenolic compounds, may provide additional chemical protection against herbivore attacks (McKey, 1979; Sudd and Sudd, 1985; Nicolson and Thornburg, 2007; Baker-Meio and Marquis, 2012; Guesdon et al., 2019). Thus, outer calyx trichomes and outer floral glands on the studied species are probably associated with attracting anti-herbivore protectors. Further morpho-anatomical and histochemical studies are required to confirm their detailed internal structures and functions.

A conical or papillose corolla epidermis is found on various angiosperm species (Christensen and Hansen, 1998; Whitney and Glover, 2007; Song et al., 2020), where its tactile cues may promote pollination by certain insects (Whitney et al., 2009, 2011). All studied species displayed a papillose cell pattern on the inner corolla. Only *L. erythrorhizon* had a unique papillose knobby pattern, which resulted in much higher and sharper pointed cells than a papillose conical type, (average papillose knobby cell height, 36.74 μm; papillose conical cell height, 10.70 μm), with non-glandular and glandular trichomes. As with the presence or absence of faucal appendages, inner corolla cellular patterns are also associated with pollinators. More comprehensive studies on differences in pollination position, visiting rate of pollinator, or pollen shape in *Lithospermum* and *Arnebia* will provide better understanding of their reproduction.

Gynoecium characteristics, such as stylar lobation or stigma number and shape in *Arnebia* and related genera, are critical for distinguishing between groups and species (Zhu et al., 1995; Ambrish and Srivastava, 2014). In particular, the degree of lobation on the style is a key characteristic to discriminate the genera *Arnebia* and *Huynhia*, as well as *Arnebia* species (Coppi et al., 2015). Here, we detected differences in stylar lobation and stigma shape between the very similar *A. guttata* and *A. tibetana* species. All *A. guttata* had a consistently 2-fid style with oblong stigmas; whereas *A. tibetana* had a 2-cleft style with oblate stigmas. Notably, all *Arnebia* species shared the same tuberculate stigma cell pattern.

### Identification Key for Species Used to Prepare the Medicine, Lithospermi Radix

Based on the floral external and epidermal micromorphological characteristics of three *Arnebia* and one *Lithospermum* species, we provide the following identification key.

1. Flower color white; calyx 5-parted; outer and inner corolla surface with papillose knobby cells; style linear, indistinct; stigma asterisk-tuberculate …………………….. *L. erythrorhizon*
1’. Flower color purple or yellow; calyx 5-lobed; outer corolla surface with tabular rugose and inner corolla with papillose conical cells; style filiform, distinct; stigma tuberculate ….2
2. Flower color purple; both outer calyx and outer corolla surfaces pubescent with smooth glandular and non-glandular trichomes; inner corolla surface glabrous; stigma globose ………………………………………………………… *A. euchroma*
2’. Flower color yellow; both outer calyx and outer corolla surfaces pubescent with verrucate non-glandular trichomes; inner corolla surface pubescent with smooth glandular trichomes with stalk; stigma not-globose ………. 3
3. Style 2-cleft; stigma oblate ……………………………….. *A. guttata*
3’. Style 2-fid; stigma oblong ………………………………. *A. tibetana*

### Characterization of cp Genome and Genetic Variations in *Arnebia* and *Lithospermum* Species

We determined the complete cp genomes of three *Arnebia* and one *Lithospermum* accessions. The organization of these four cp genomes follows that of other Boraginaceae (Chen and Zhang, 2019; Guo et al., 2020), and includes a typical quadripartite structure with LSC and SSC regions separated by two IRs. Genomes in the tested *Arnebia* and *Lithospermum* species possess 112 unique genes. Their gene order, GC content, genomic structure, and overall length (149,316–150,465 bp) conform to previously described angiosperm cp genomes (Millen et al., 2001). Codon usage is a key factor for correct expression of genetic information, and plays an important role in shaping cp genome evolution (Wang et al., 2016; Yi et al., 2018). *Arnebia* and *Lithospermum* accessions possess nearly identical codons, which are similar to those of other cp genomes (Wang et al., 2016; Raman et al., 2019). RSCU values indicated synonymous codon usage bias, with a disproportionate number of codons having an A or T in the third position. This phenomenon has been described in many angiosperm cp genomes and is consistent with the pattern in seed plants. Indeed, high RSCU values correspond to more highly conserved cp genes (Wang et al., 2016; Ivanova et al., 2017; Zuo et al., 2017). We surveyed the RSCU values for six Boraginaceae cp genomes downloaded from NCBI. Half of the codons had a weak codon bias (Supplementary Figure 4), denoted in green in the figure (RSCU > 1). This result is similar to other cp genomes, and most codons with high RSCU values had an A or T in the third position. The cp genome of *B. officinalis* displayed slightly different codon bias than other Boraginaceae species. In particular, GGT (Guanine), AGG (Arginine), and GGG (Guanine) codons had relatively low RSCU values; whereas TGT (Cysteine) and CGA (Arginine) had somewhat higher RSCU values.

The mVISTA results show that the cp genomes of *Arnebia* and *Lithospermum* possess relatively high diversity, with genic regions being more conserved than intergenic spacer regions, as is typical in angiosperm cp genomes (Shaw et al., 2007; Huo et al., 2019; Song J. H. et al., 2019). Even though the cp genomes of *A. guttata* and *A. tibetana* displayed closer proximity than other cp genomes, *trnS-GCU-trnG-UCC*, *trnC-GCA-petN*, *psaA-ycf3*, *trnI-CAU-ycf2*, *psbE-petL, clpP*, and *rps15-ycf1* regions were found to be hotspots of genetic variation (Figure 4). Hotspot regions in plants indicate underlying evolution (Morton and Clegg, 1993; Maier et al., 1995; Liu H. et al., 2018; Liu H. et al., 2019) and can be used to distinguish species or genera. Molecular markers relative to these regions serve for the identification of valuable herbal medicinal plants (Cho et al., 2015; Hong et al., 2017; Park et al., 2018a,b). In terms of nucleotide diversity, most divergent regions were non-coding, which is again consistent with other cp genomes (Schroeder et al., 2016; Zhitao et al., 2017; Liu E. et al., 2019; Smidt et al., 2020). Other cp genomes were reported to have highly variable non-coding regions at *trnS-GCU-trnG-UCC*, *trnC-GCA-petN*, *psaA-ycf3*, *trnI-CAU-ycf2*, *psbE-petL*, and *rps15-ycf1*. We noticed higher diversity in the *atpB-rbcL* region between *A. guttata* and *A. tibetana* compared to the other four species. This divergence indicated a species-unique region, which could be clearly distinguished and used for identification.

Usually, angiosperm cp genomes are highly conserved in length, structure, gene number, and gene order (Jansen and Ruhlman, 2012). IR contraction and expansion cause the length of the genome to vary (Raubeson et al., 2007). Here, we compared six Boraginaceae cp genomes, including *Arnebia* and *Lithospermum*, against *F. suspensa* (family Oleaceae) as reference (Supplementary Figure 5). The six Boraginaceae cp genomes were well conserved in terms of gene order and positions. Moreover, except for *T. plicata*, they all indicated IR contraction. Specifically, *rps2* was located in LSC/IRa and *ycf1* was identified in the IRa/SSC junction, which overlapped with *ndhF*. These findings revealed the occurrence of IR contraction and expansion, as well as their effect on genome length.

Most genes in the Boraginaceae exhibited stable gene evolution (purifying selection). Positive selection was detected in *rbcL*, a gene encoding the large subunit of Rubisco, which is commonly observed in angiosperm cp genomes due to the elevated selective pressure for this protein (Kapralov and Filatov, 2007; Shi et al., 2019; Yao et al., 2019). Large-scale studies confirmed that positive selection of *rbcL* is widespread among land plants. Thus, Kapralov and Filatov (2007) evaluated positive selection in *rbcL* in over 3,000 species. and reported positive selection in most land plants but not in algae and cyanobacteria. A similar study supported the positive selection of *rbcL* in *Ilex* using 240 *Ilex* sequences (Yao et al., 2019). Positive selection in the chloroplast genome, including at *rbc*L, has been reported in numerous plant taxa (Bock et al., 2014). The results of our study corroborated the results of the previous studies. The positive selection suggests ongoing adaptation to the environment (Kimura, 1989; Raman and Park, 2016; Ivanova et al., 2017).

Taken together, we demonstrated that Boraginaceae chloroplast genomes are similar to other angiosperm genomes, whereas sequence divergence of the chloroplast genome was identified between *A. guttata* and *A. tibetana* (Figures 6, 7). Previously, species identification of *A. guttata* and *A. tibetana* based on only general morphological characterization was difficult, and their taxonomic classification was also unclear. However, we found the noticed evidence for distinguishing this group. The complete sequences of *Arnebia* chloroplast genomes enabled accurate comparative analysis and the identification of species-specific traits for accurate classification of *Arnebia* species.

### Development of Molecular Markers for the Identification of Medicinal Plants

Artaxa guttata, *A. euchroma*, and *L. erythrorhizon* are valuable medicinal plants in traditional Korean herbal medicine (Korea Institute of Oriental Medicine [KIOM], 2020). To ensure quality control, safety, and effectiveness of their ingredients, Herbal Pharmacopeia mandates that herbal medicinal materials should be kept in pure form. Adulterants may cause negative side effects and quality problems. The purity of herbal medicinal ingredients can be tested with species-specific molecular markers. Here, we developed indel marker sets to distinguish between four species of medicinal Boraginaceae. Although cp genomes are highly conserved within the same genus, they frequently possess insertion/deletion regions. We detected the conserved indel regions *psaA*- *ycf3*, *trnI-CAU- ycf2*, and *ndhC*-*trnV-UAC*. The corresponding marker sets *psaA*- *ycf3* (PSY), *trnI-CAU- ycf2* (TCY), and *ndhC*-*trnV-UAC* (NCTV) successfully discriminated between the four species, as well as between the closely related *A. guttata* and *A. tibetana*. The rapid and accurate species identification achieved by these marker sets will help distinguish *Arnebia* and *Lithospermum* without having to rely on morphological analysis, and will prevent incorrect classification of ambiguous samples from valuable *Arnebia* species.

### Phylogenetic Relationships Among Boraginaceae and Taxonomic Identity of *Arnebia tibetana*

Complete cp genomes provide sufficient information to study the phylogenetic relationships among plants, including at low taxonomy level and for unresolved taxa (Jansen et al., 2007; Park et al., 2019, 2020). ITS and cp loci have confirmed the correct classification of numerous Boraginaceae species and inference of their evolutionary relationships based on morphological characteristics (Cohen, 2014). However, the *Arnebia* genus maintains an ambiguous phylogenetic position with respect to other monophyletic species and genera, such as *Onosma*, *Cerinthe*, *Moltkia*, and *Cystostemon*. This phylogenetic relationship derives from the use of only two *Arnebia* species out of 224 Boraginaceae. The monophyletic relationship between previously recognized clades and the position of various unplaced genera in Boraginaceae have been reclassified (Chacón et al., 2016); however, infrageneric classification of *Arnebia* species has remained unresolved (Coppi et al., 2015). Although it comprises only 25–30 species, the genus *Arnebia* is valuable in traditional herbal medicine. Here, we attempted to determine phylogenetic relationships within Boraginaceae using cp genomes. ML and BI values based on the whole chloroplast genome and CDS dataset offered powerful evidence (Figure 9) of clear species clustering within families according to the APG IV classification system. In Boraginaceae, *A. guttata* and *A. tibetana* were found to group monophyletically with a sister group, *A. euchroma*, which is consistent with previous findings (Wu et al., 2017; Liu J. et al., 2020).

Since *A. tibetana* was firstly described by Kurz (1874), this species has been regarded as a synonym of *A. guttata* (Kazmi, 1970; Ambrish and Srivastava, 2014; Urgamal et al., 2014) or as an independent species (Zhu et al., 1995; Lazkov and Sultanova, 2014). In Flora of China, *A. tibetana* is placed as a subspecies owing to its narrow distribution in Xizang, Xinjiang in China, and adjacent Xinjiang in Kyrgyzstan and its absence of blackish purple spots on corolla lobes (Zhu et al., 1995). Moreover, Lazkov and Sultanova (2014) recognized and listed *A. guttata* and *A. tibetana* as independent species in the Flora of Kyrgyzstan on the basis of the different life cycles (*A. guttata*, perennial; *A. tibetana*, biennial). During our field survey, we found that the flowers with or without blackish purple spots on corolla lobes in both *A. guttata* and *A. tibetana*. The blackish purple spots appear during the flowering season, but disappear after pollination and at withering in *A. guttata* and *A. tibetana*. Thus, this traditional diagnostic character for distinguishing *A. tibetana* (Zhu et al., 1995) is not useful because they are the seasonal variation. However, based on our consistently observed floral morphological characteristics, such as style lobation, and stigma shape, and genetic distance of cp genomes reported in this study, *A. tibetana* is recognized as an independent species following Kurz (1874)’s taxonomic concept with newly found diagnostic characteristics, 2-fid style and oblong stigma.

## Conclusion

Floral morphological and micromorphological characteristics, as well as chloroplast (cp) genome sequences of selected species in *Arnebia* and *Lithospermum* genera were analyzed. The floral characteristics reveal great diversity in floral epidermis cell patterns, gynoecium, and structure of trichomes. Chloroplast genomes of the four species were well conserved with respect to genome length, gene number, gene orientation, and local divergence. Most variable sequences were detected in non-coding regions. These hotspots were used to develop new indel molecular marker sets for efficient and rapid species identification. We successfully established phylogenetic relationships among *Arnebia* species in Boraginaceae using cp genome sequences. Accordingly, we suggest that *A. tibetana* be recognized as an independent species following Kurz (1874)’s taxonomic concept based on its narrow distribution, different life cycle, and newly found gynoecium characteristics and genetic distance of cp genomes in this study. Morphological characteristics, genomic data, and taxonomic concept presented in our study for medicinal *Arnebia* and *Lithospermum* species will be helpful in further research on Boraginaceae.

## Data Availability Statement

The datasets presented in this study can be found in online repositories. The names of the repository/repositories and accession number(s) can be found below: https://www.ncbi. nlm.nih.gov/genbank/, MT975391; https://www.ncbi.nlm.nih.gov/genbank/, MT975392; https://www.ncbi.nlm.nih.gov/genbank/, MT975393; https://www.ncbi.nlm.nih.gov/genbank/, MT975394; https://datadryad.org/stash, https://doi.org/10.5061/dryad.gtht76hk7.

## Author Contributions

IP and J-HS designed the experimental framework and drafted and revised the manuscript. J-HS, SY, and BM collected and identified plant materials. J-HS performed microscopic analysis, whereas IP performed molecular experiments and carried out genome analysis. IP, J-HS, and BM revised the manuscript. All authors contributed to the experiments and approved the final manuscript.

## Conflict of Interest

The authors declare that the research was conducted in the absence of any commercial or financial relationships that could be construed as a potential conflict of interest.

## Footnotes

1 https://www.geneious.com
