# Supplementary material for: Dissection for Floral Micromorphology and Plastid Genome of Valuable Medicinal Borages Arnebia and Lithospermum (Boraginaceae)
Source: Front Plant Sci. 2020 Dec 4;11:606463. doi: 10.3389/fpls.2020.606463 (PMC7746654; doi:10.3389/fpls.2020.606463)
Supplement: Supplementary file 1 [file Table_1.DOCX]

Supplementary Material

**Dissection for floral micromorphology and plastid genome of valuable medicinal borages *Arnebia* and *Lithospermum* (Boraginaceae)**

Inkyu Park, Sungyu Yang, Jun-Ho Song*, Byeong-Cheol Moon*

***Correspondence:**

Jun-Ho Song: songjh@kiom.re.kr

Byeong Cheol Moon: bcmoon@kiom.re.kr

# Supplementary Figures and Tables
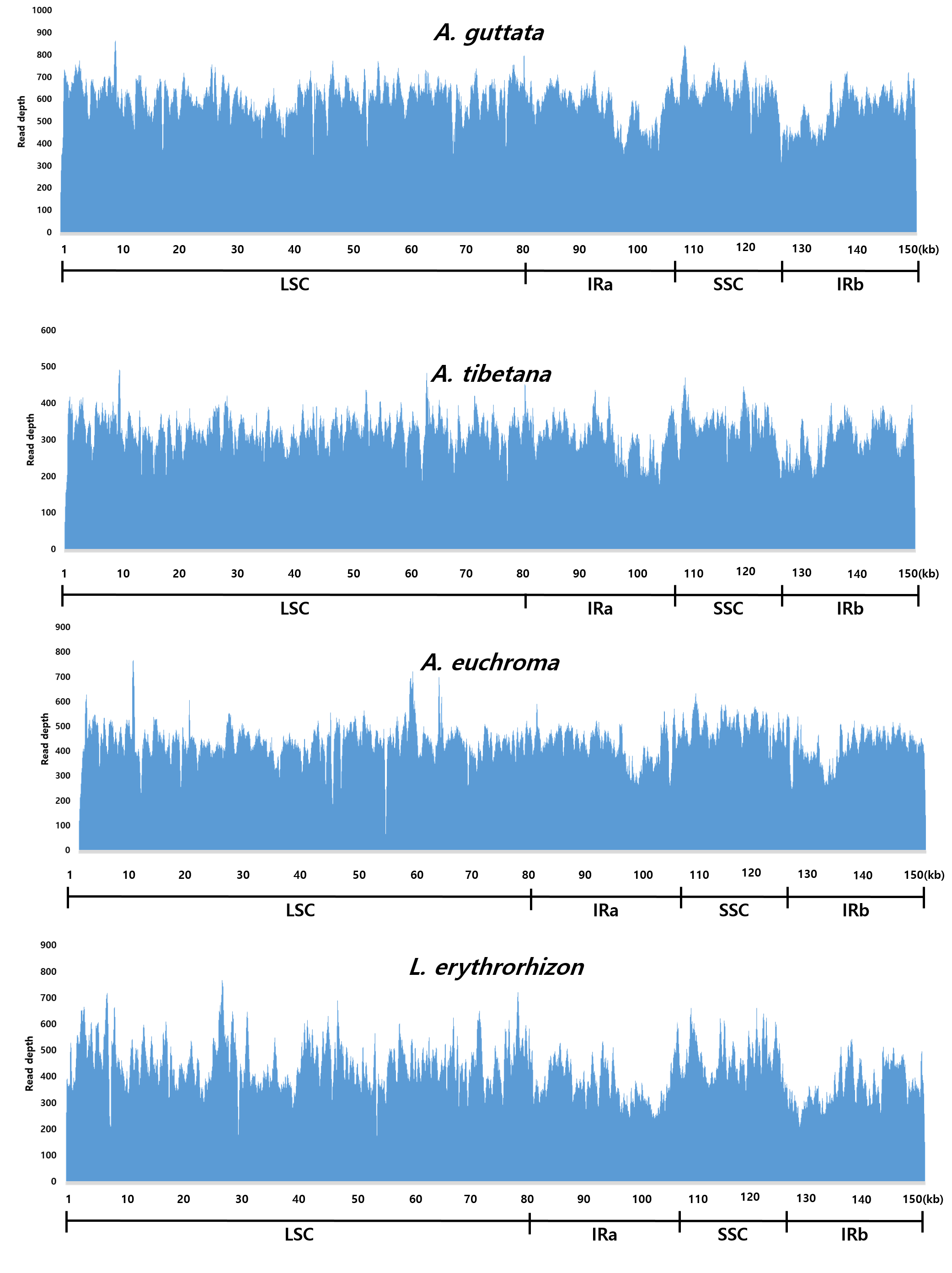
Figure S1 Distribution of paired-end reads mapped onto complete chloroplast genomes of three *Arnebia* and one *Lithospermum* species. LSC, large single-copy region; SSC, small single-copy region; IRa, inverted repeat a; IRb, inverted repeat b.


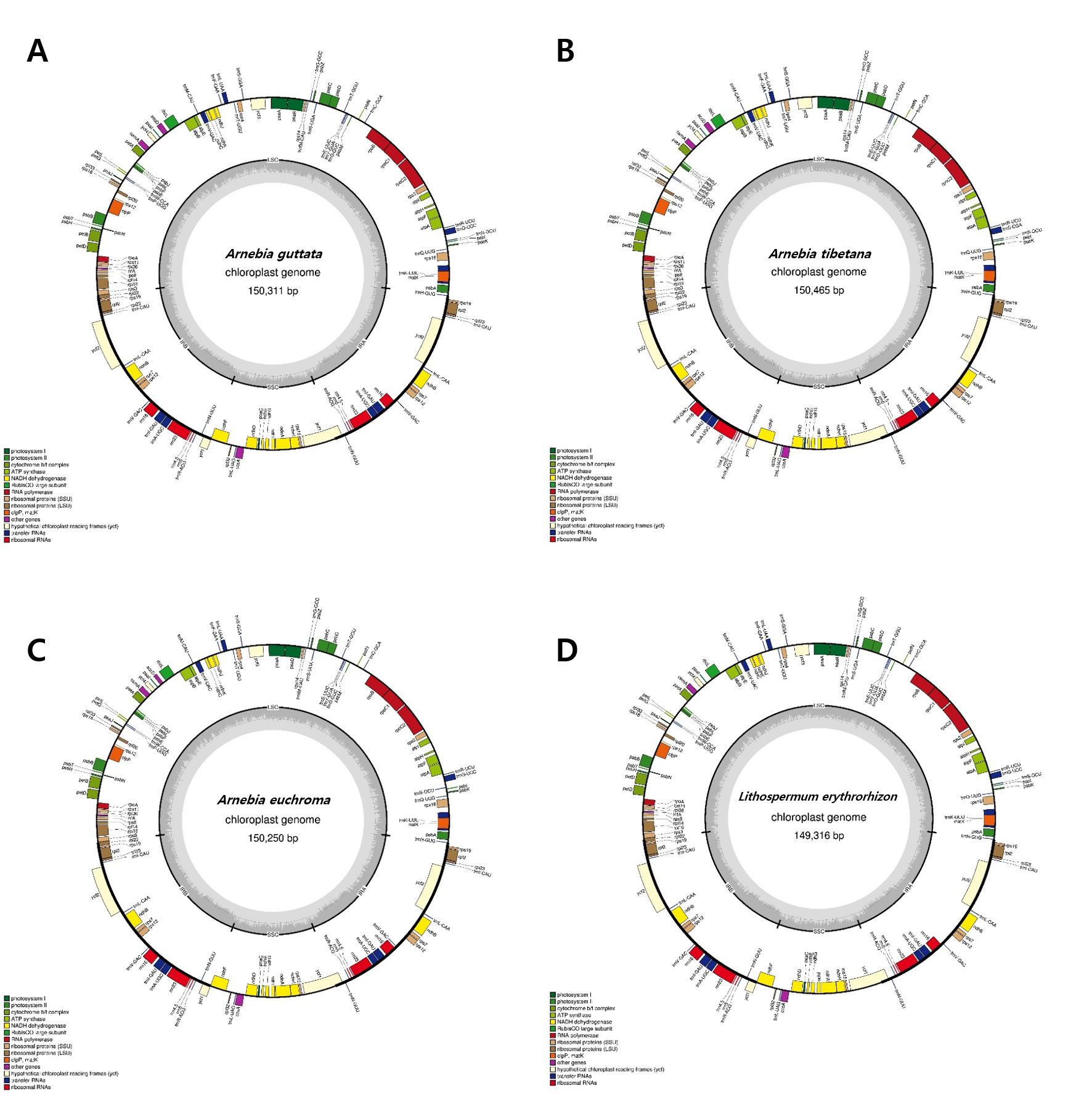


Figure S2 Circular gene map of chloroplast genomes from *Arnebia* and *Lithospermum* species. (A) *A. guttata*, (B) *A. tibetana*, (C) *A. euchroma*, (D) *L. erythrorhizon.*


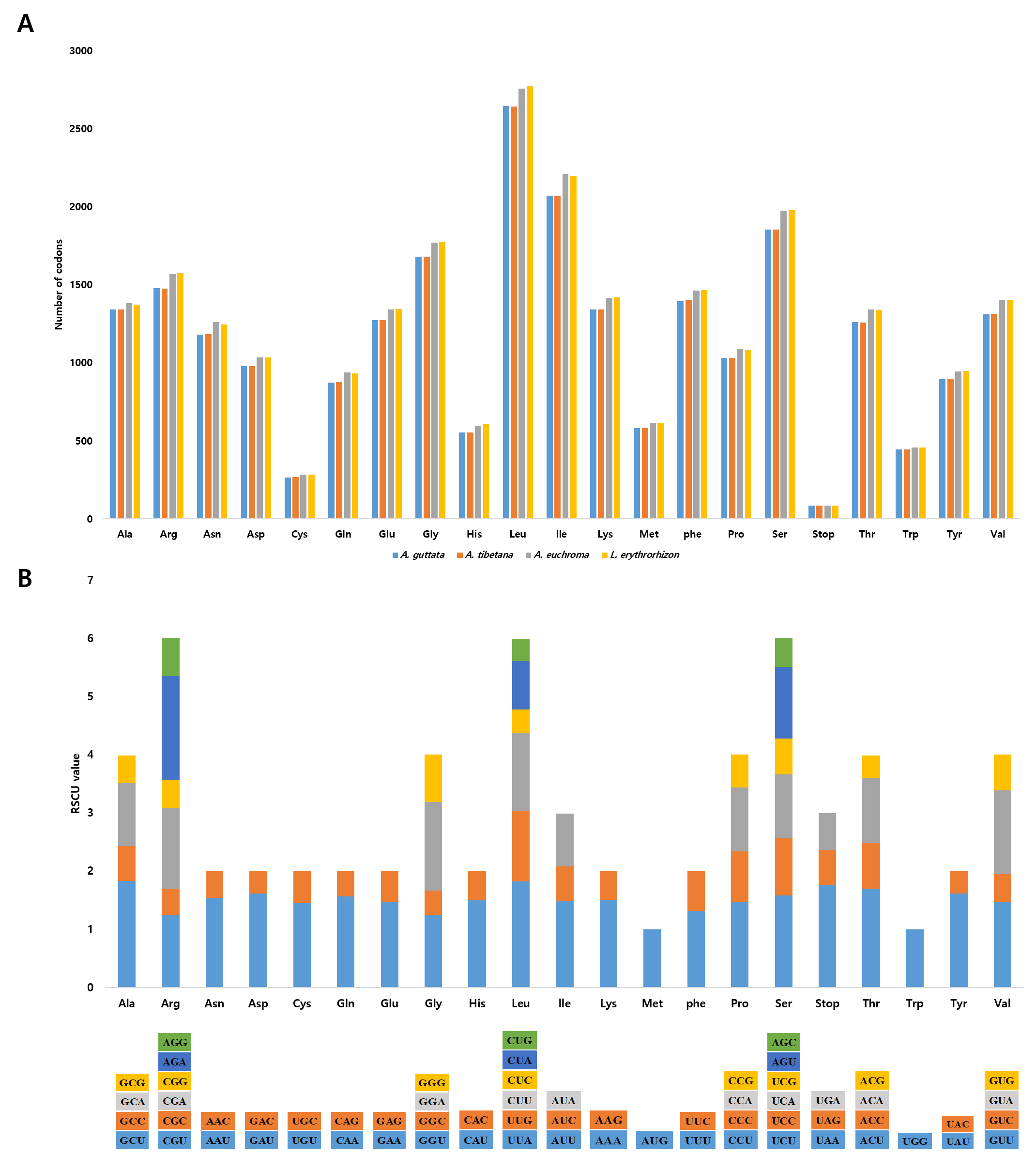


Figure S3 Codon frequencies and RSCU values for three *Arnebia* and one *Lithospermum* chloroplast genome. (A) Amino acid frequencies for protein-coding sequences. (B) Codon usage for 20 amino acids and stop codons in 78 protein-coding genes.

**
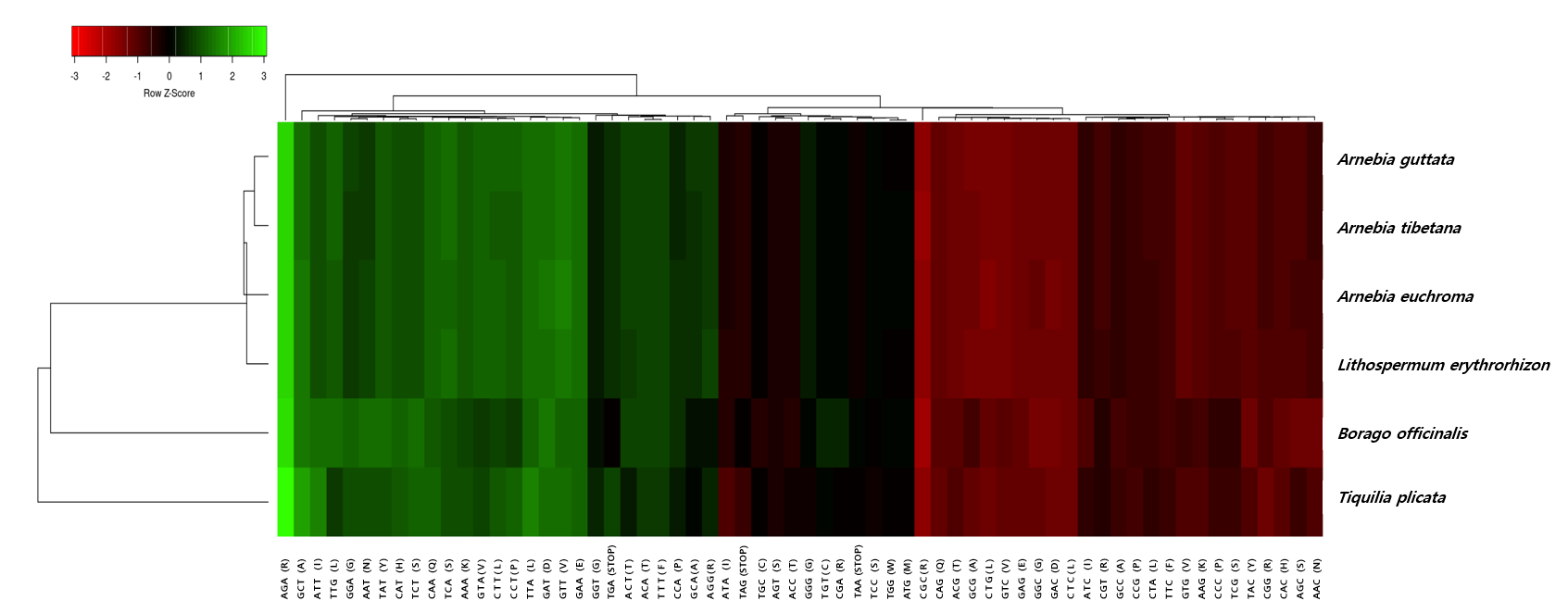
**

Figure S4 Codon distribution of protein-coding genes in Boraginaceae chloroplast genomes. Green indicates a high relative synonymous codon usage (RSCU) value and red indicates a low RSCU value. Hierarchical clustering (average linkage method) was performed based on the codon patterns (x-axis).


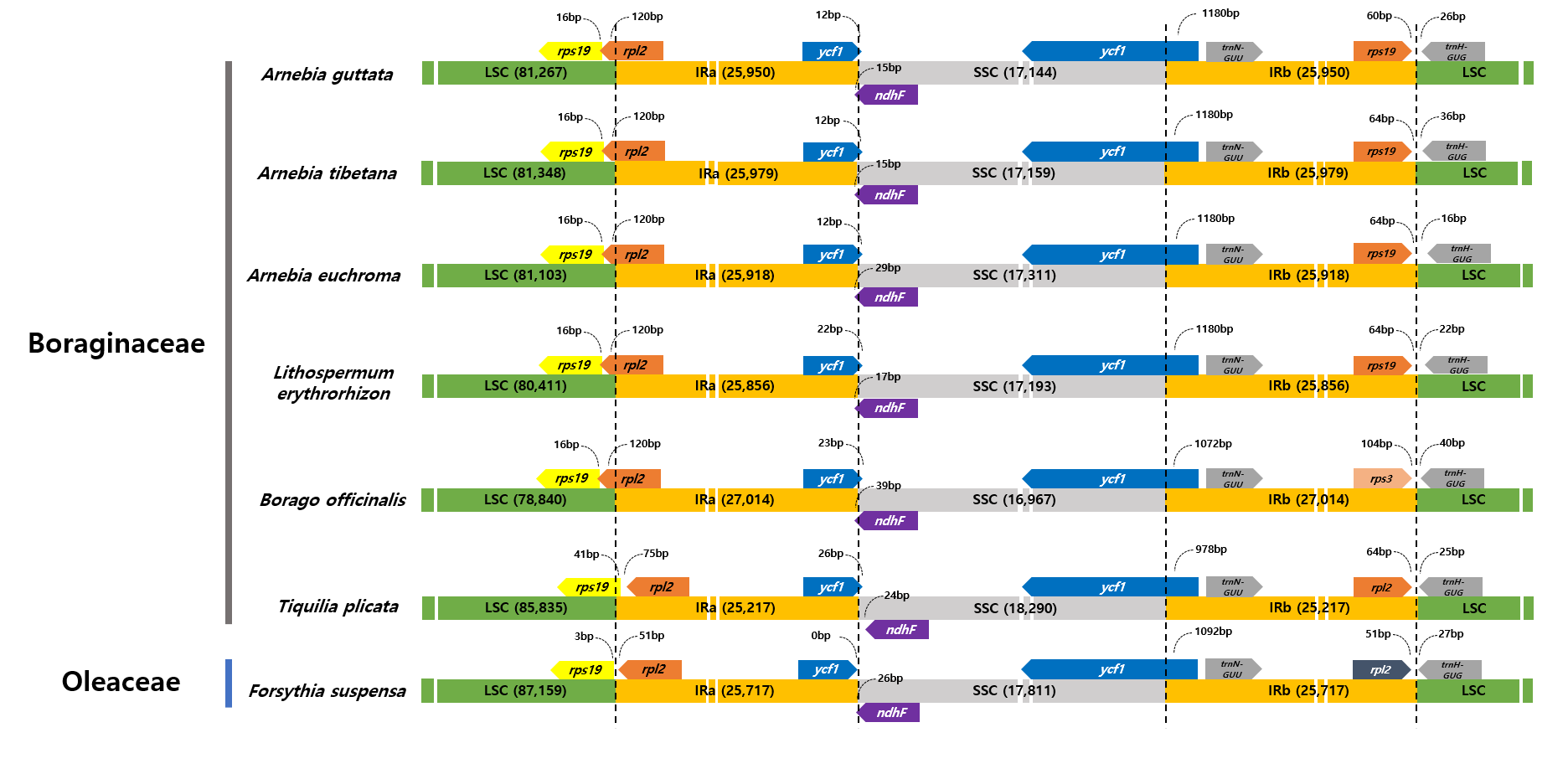


Figure S5 Comparison of LSC, IR, and SSC junction positions among Boraginaceae chloroplast genomes. LSC, large single-copy region; SSC, small single-copy region; IR, inverted repeat.


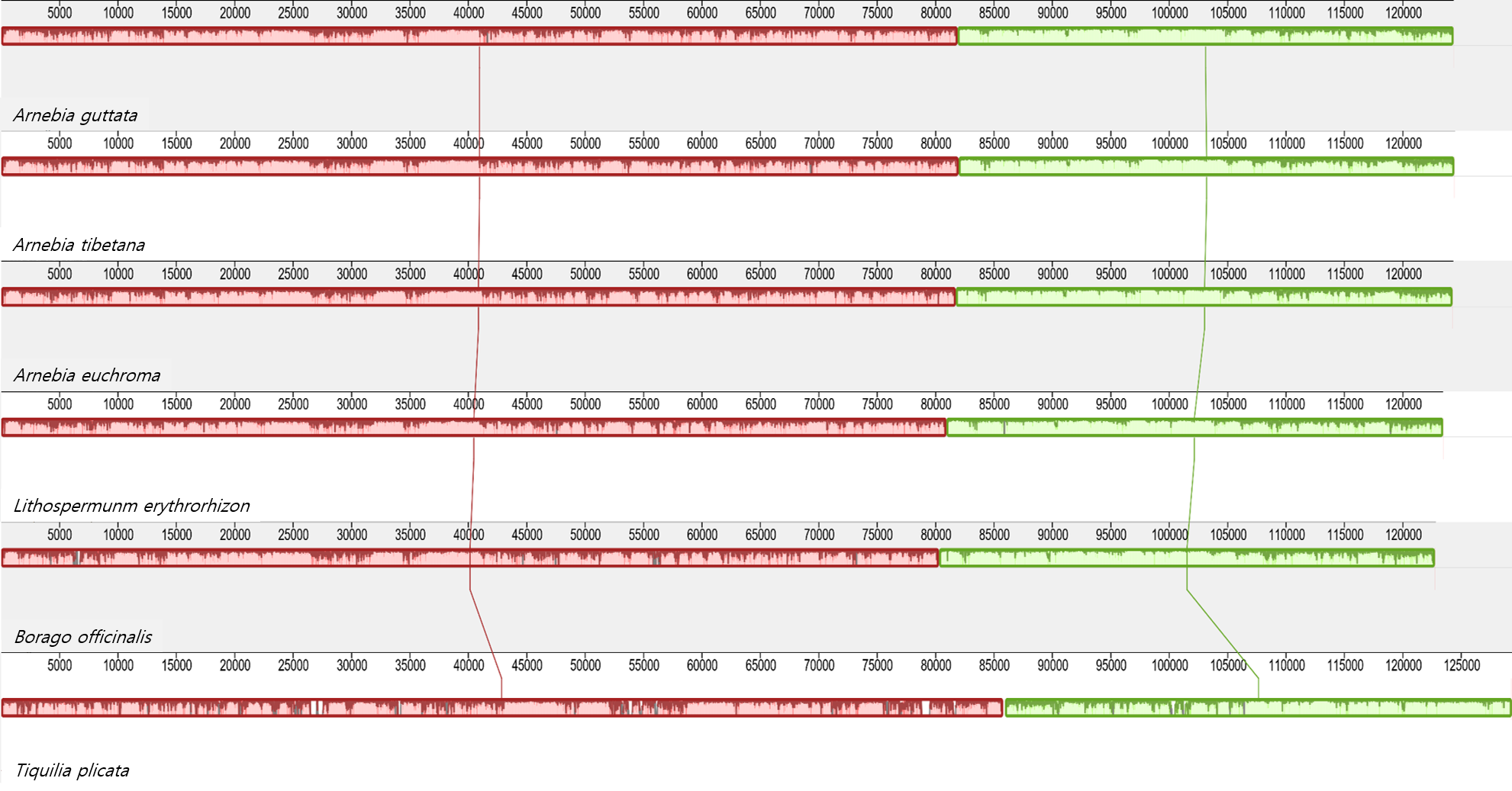


Figure S6 Comparison of complete cp genomes from six Boraginaceae species using the MAUVE algorithm.


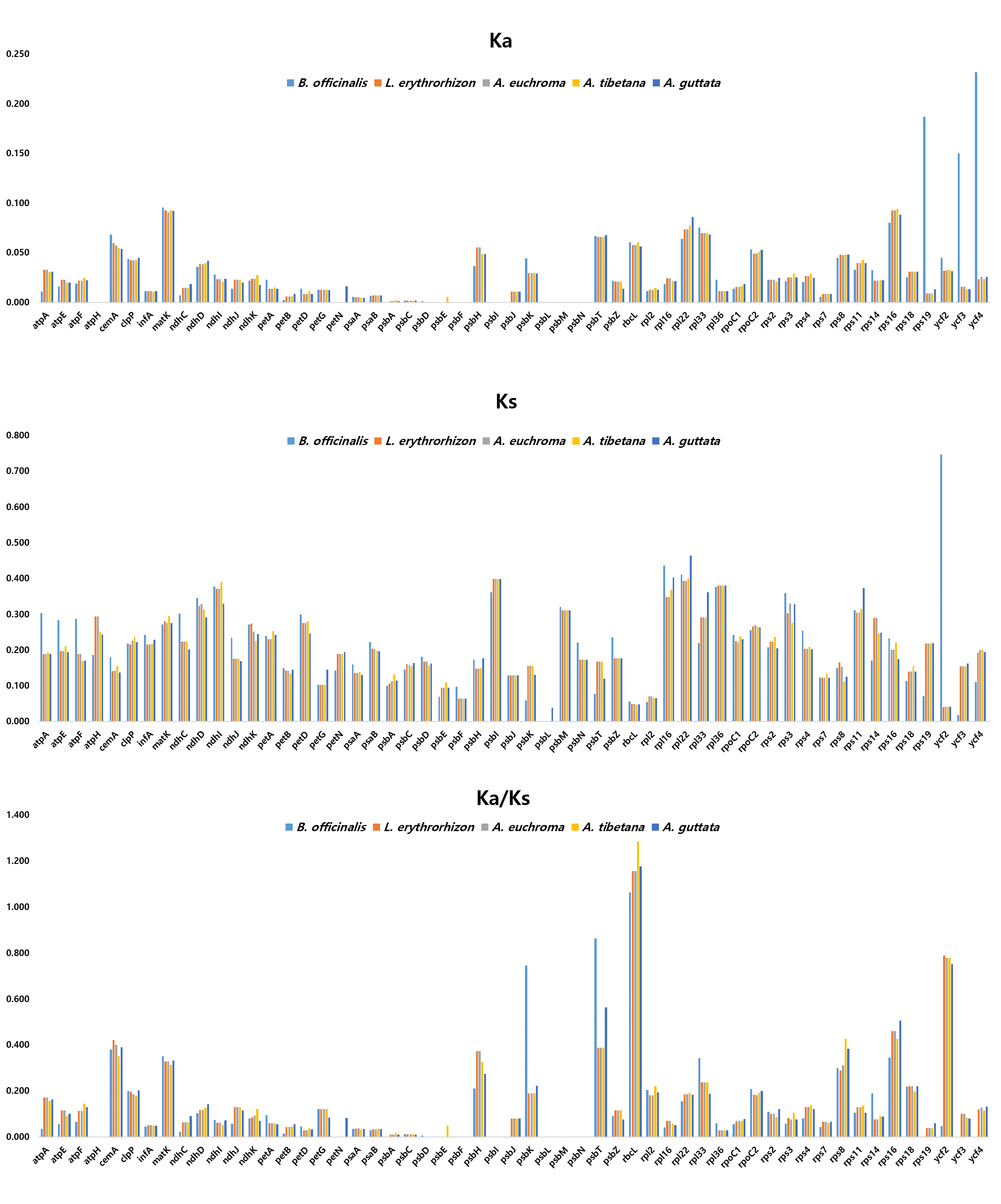


Figure S7 Ka and Ks values for Boraginaceae cp genomes. The ratios of non-synonymous substitution (Ka) to synonymous substitution (Ks) were calculated for 77 conserved protein-coding sequences using *Borago officinalis* as reference. Genes with Ka or Ks = 0 are not shown. Ka and Ks values are shown in green and blue, respectively.


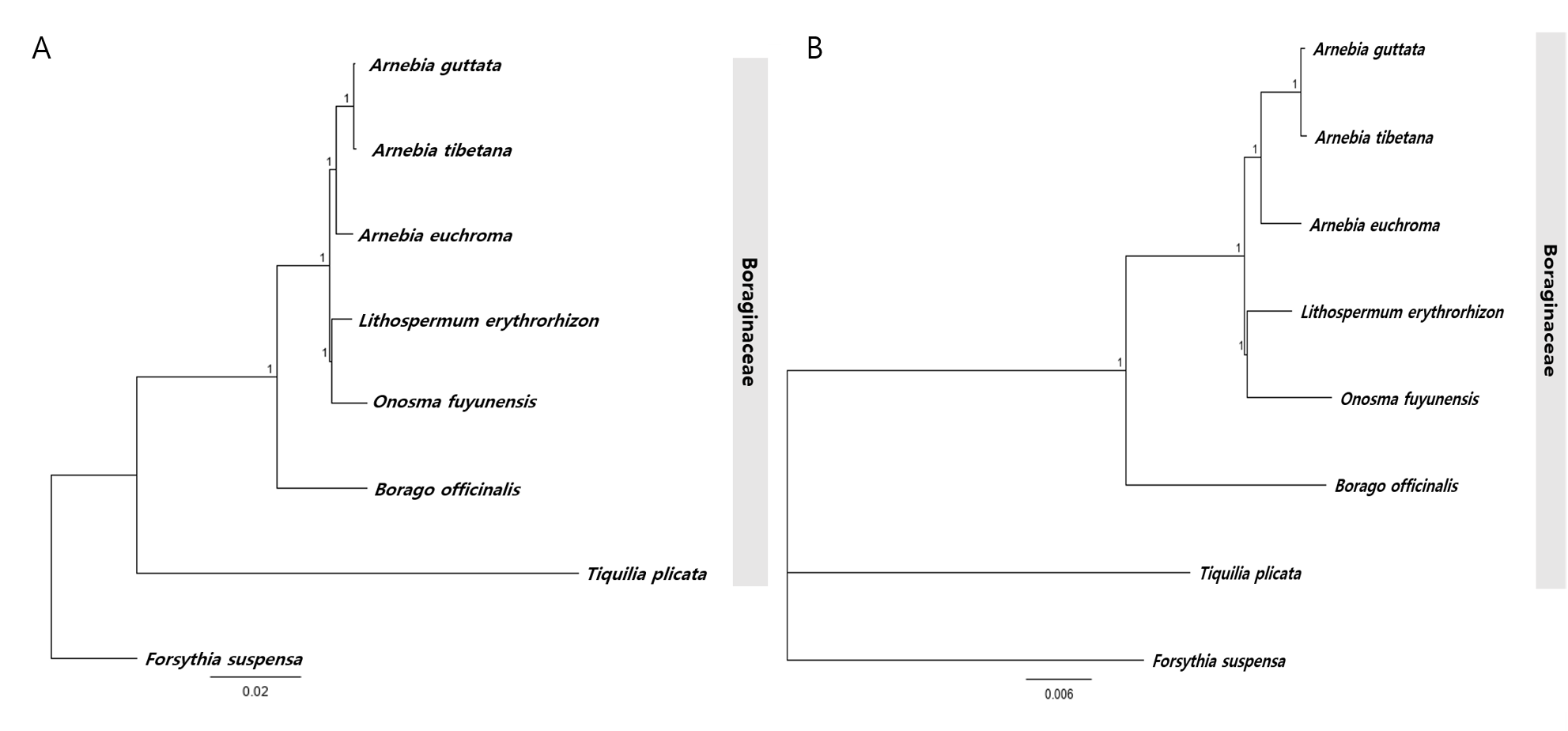


**Figure S8 Phylogenetic tree based on whole chloroplast genome (A) and 71 protein-coding genes (B) from four *Arnebia* and one *Lithospermum* species using Bayesian posterior probabilities.** Posterior probabilities are indicated next to each branching point.

**Table S1 Characteristics of the floral morphology and chloroplast genomes used in this study.**

| No. | Species | Collection information | Coordinates | Voucher number |
| --- | --- | --- | --- | --- |
| 1 | *Arnebia guttata* Bunge | Khanbogd sum, Umnugobi province, Mongolia | 43°11'09.4"N 107°10'48.0"E | KIOM201901022035^G,M^ |
| 2 |  | Bulgan sum, Khovd province, Mongolia | 46°04'16.8"N 91°10'54.4"E | KIOM201901022034^M^ |
| 3 | *Arnebia tibetana* Kurz. | Uzonggu Kuush, Jeti-Ögüz District, Issyk-Kul Region, Kyrgyzstan | 41°17'41.7"N 78°04'34.2"E | KIOM201901022438^G,M^ |
| 4 |  | Uzonggu Kuush, Jeti-Ögüz District, Issyk-Kul Region, Kyrgyzstan | 41°17'41.7"N 78°04'34.2"E | KIOM201901022437^M^ |
| 5 |  | Uzonggu Kuush, Jeti-Ögüz District, Issyk-Kul Region, Kyrgyzstan | 41°17'41.7"N 78°04'34.2"E | KIOM201901022436^M^ |
| 6 |  | Uzonggu Kuush, Jeti-Ögüz District, Issyk-Kul Region, Kyrgyzstan | 41°17'41.7"N 78°04'34.2"E | KIOM201901022435^M^ |
| 7 |  | Uzonggu Kuush, Jeti-Ögüz District, Issyk-Kul Region, Kyrgyzstan | 41°17'41.7"N 78°04'34.2"E | KIOM201901022434^M^ |
| 8 |  | Engilchek, Ak-Suu District, Issyk-Kul Region, Kyrgyzstan | 42°14'39.5"N 79°06'53.9"E | KIOM201901022431^M^ |
| 9 |  | Engilchek, Ak-Suu District, Issyk-Kul Region, Kyrgyzstan | 42°14'39.5"N 79°06'53.9"E | KIOM201901022430^M^ |
| 10 |  | Engilchek, Ak-Suu District, Issyk-Kul Region, Kyrgyzstan | 42°14'39.5"N 79°06'53.9"E | KIOM201901022429^M^ |
| 11 |  | Engilchek, Ak-Suu District, Issyk-Kul Region, Kyrgyzstan | 42°14'39.5"N 79°06'53.9"E | KIOM201901022428^M^ |
| 12 |  | Engilchek, Ak-Suu District, Issyk-Kul Region, Kyrgyzstan | 42°14'39.5"N 79°06'53.9"E | KIOM201901022427^M^ |
| 13 | *Arnebia euchroma* (Royle) I.M. Johnst | Uzonggu Kuush, Jeti-Ögüz District, Issyk-Kul Region, Kyrgyzstan | 41°17'21.3"N 78°04'33.0"E | KIOM201901022425^G,M^ |
| 14 |  | Uzonggu Kuush, Jeti-Ögüz District, Issyk-Kul Region, Kyrgyzstan | 41°17'21.3"N 78°04'33.0"E | KIOM201901022424^M^ |
| 15 |  | Uzonggu Kuush, Jeti-Ögüz District, Issyk-Kul Region, Kyrgyzstan | 41°17'21.3"N 78°04'33.0"E | KIOM201901022423^M^ |
| 16 |  | Uzonggu Kuush, Jeti-Ögüz District, Issyk-Kul Region, Kyrgyzstan | 41°17'21.3"N 78°04'33.0"E | KIOM201901022422^M^ |
| 17 |  | Uzonggu Kuush, Jeti-Ögüz District, Issyk-Kul Region, Kyrgyzstan | 41°17'21.3"N 78°04'33.0"E | KIOM201901022421^M^ |
| 18 |  | Uzonggu Kuush, Jeti-Ögüz District, Issyk-Kul Region, Kyrgyzstan | 41°17'21.3"N 78°04'33.0"E | KIOM201901022420^M^ |
| 19 |  | Uzonggu Kuush, Jeti-Ögüz District, Issyk-Kul Region, Kyrgyzstan | 41°17'21.3"N 78°04'33.0"E | KIOM201901022419^M^ |
| 20 |  | Uzonggu Kuush, Jeti-Ögüz District, Issyk-Kul Region, Kyrgyzstan | 41°17'21.3"N 78°04'33.0"E | KIOM201901022418^M^ |
| 21 |  | Uzonggu Kuush, Jeti-Ögüz District, Issyk-Kul Region, Kyrgyzstan | 41°17'21.3"N 78°04'33.0"E | KIOM201901022417^M^ |
| 22 |  | Uzengu-kuush river basin, Jety – Oguz, Kyrgyzstan | 41°17'21.3"N 78°04'33.0"E | KIOM201901022425^M^ |
| 23 | *Lithospermum erythrorhizon* Siebold & Zucc. | Cheongsong-gun, Gyeongsangbuk-do, Korea | 41°19'27.2"N 78°04'59.5"E | KIOM201501011555^G,M^ |
| 24 |  | Pohang-si, Gyeongsangbuk-do, Korea | 36°15'47.1"N 129°21'42.7"E | KIOM201801020633^M^ |

^G^, chloroplast genomic study; ^M^, floral morphology.

**Table S2 Primers used in this study for chloroplast junction validation.**

| Primer name | Primer sequence (5`>3`) | Position |
| --- | --- | --- |
| LI_F | CCCCGCGGACGACTTTCTAT | LSC_IRa |
| LI_R | CGAGGACATGCCAAAAGCG |  |
| IS_F | CAGCTTCCGAAATGAGGGGG | IRa_SSC |
| IS_R | GCGGGCCTTTTATTAGTATTGCC |  |
| SI_F | GGGAATCTGTATACGGAAGAAGGA | SSC_IRb |
| SI_R | AGAAAGGGAGGATCCGGACA |  |
| IL_F | CGAGGACATGCCAAAAGCGA | IRb_LSC |
| IL_R | ACTTAGCTGCTGTCGAAGCTC |  |

**Table S3 PCR-based sequence validation of chloroplast junctions.**

| Species | Location | PCR-based sequence (bp) | Start | End | Identities (%) | No. |
| --- | --- | --- | --- | --- | --- | --- |
| *A. guttata* | LSC_IRa | 840 | 80,892 | 81,732 | 100 |  |
|  | IRa_SSC | 940 | 106,934 | 107,873 | 100 |  |
|  | SSC_IRb | 779 | 123,788 | 124,566 | 100 |  |
|  | IRb_LSC | 907 | 149,848 | 443 | 100 |  |
| *A. tibetana* | LSC_IRa | 840 | 80,973 | 81,812 | 100 |  |
|  | IRa_SSC | 940 | 107,038 | 107,977 | 100 |  |
|  | SSC_IRb | 785 | 123,913 | 124,697 | 100 |  |
|  | IRb_LSC | 893 | 150,002 | 429 | 100 |  |
| *A. euchroma* | LSC_IRa | 840 | 80,728 | 81,566 | 100 |  |
|  | IRa_SSC | 932 | 106,744 | 107,675 | 100 |  |
|  | SSC_IRb | 773 | 123,759 | 124,531 | 100 |  |
|  | IRb_LSC | 907 | 149,788 | 444 | 100 |  |
| *L. erythrorhizon* | LSC_IRa | 840 | 79,963 | 80,801 | 100 |  |
|  | IRa_SSC | 925 | 105,988 | 106,912 | 100 |  |
|  | SSC_IRb | 773 | 122,889 | 123,661 | 100 |  |
|  | IRb_LSC | 793 | 148,927 | 403 | 100 |  |

**Table S4 Characteristics of the plant samples used for developing new indel markers.**

| No. |  | Species | Collection information | Coordinates | Voucher number |
| --- | --- | --- | --- | --- | --- |
| 1 | 1 | *A. guttata* | Jeti-Ögüz District, Issyk-Kul Region, Kyrgyzstan | 41°17'41.7"N 78°04'34.2"E | KIOM201901022438 |
| 2 | 2 | *A. tibetana* | Khanbogd sum, Umnugobi province, Mongolia | 43°11'09.4"N 107°10'48.0"E | KIOM201901022035 |
| 3 |  |  | Jeti-Ögüz District, Issyk-Kul Region, Kyrgyzstan, | 42°14'39.5"N 79°06'53.9"E | KIOM201901022427 |
| 4 |  |  | Jeti-Ögüz District, Issyk-Kul Region, Kyrgyzstan | 42°14'39.5"N 79°06'53.9"E | KIOM201901022426 |
| 5 | 3 | *A. euchroma* | Jeti-Ögüz District, Issyk-Kul Region, Kyrgyzstan | 41°17'21.3"N 78°04'33.0"E | KIOM201901022425 |
| 6 |  |  | Jeti-Ögüz District, Issyk-Kul Region, Kyrgyzstan | 41°17'21.3"N 78°04'33.0"E | KIOM201901022424 |
| 7 | 4 | *L. erythrorhizon* | Cheongsong-gun, Gyeongsangbuk-do, Korea | 36°23'48.0"N 129°08'09.8"E | KIOM201501011555 |
| 8 |  |  | Pohang-si, Gyeongsangbuk-do, Korea | 36°15'47.1"N 129°21'42.7"E | KIOM201801020633 |

Table S5 Primer information for indel markers PSY, TCY, and NCTV.

| Primer name | Primer sequence (5`>3`) | Position |
| --- | --- | --- |
| PSY | GGGGCAAGTGTTCGGATCTA | *psaA-ycf3* |
|  | CTGAAATTGCGGAGGCTTGG |  |
| TCY | ATGAGTTGGGCGCTTTAACC | *trnI-CAU-ycf2* |
|  | TGCTTGTTGAAGCCCGTGA |  |
| NCTV | AAGACCATTCCAACGCTCCC | *ndhC-trnV-UAC* |
|  | GGTTCGAGTCCGTATAGCCC |  |

**Table S6 Chloroplast genomes from NCBI used for phylogenetic analysis.**

| No. | Family | Taxon | GenBank accession number |
| --- | --- | --- | --- |
| 1 | Boraginaceae | *Arnebia guttata* | MT975391 |
| 2 |  | *Arnebia tibetana* | MT975392 |
| 3 |  | *Arnebia euchroma* | MT975393 |
| 4 |  | *Lithospermum erythrorhizon* | MT975394 |
| 5 |  | *Borago officinalis* | NC_046796 |
| 6 |  | *Onosma fuyunensis* | NC_049569 |
| 7 | Ehretioideae^1^ | *Tiquilia plicata* | MG573056 |
| 8 | Oleaceae | *Forsythia suspensa* | NC_036367 |

^1^ Subfamily of Boraginaceae.

Table S7 Best-fitting substitution model selection using jModelTest.

| Whole cp genome data set | Model | | f(a) | f(c) | f(g) | | f(t) | | kappa | titv | Ra | | Rb | Rc | Rd | | Re | Rf | | pInv | gamma |
| --- | --- | --- | --- | --- | --- | --- | --- | --- | --- | --- | --- | --- | --- | --- | --- | --- | --- | --- | --- | --- | --- |
| AIC | GTR+I+G | | 0.3 | 0.17 | 0.2 | | 0.31 | | 3.87 | 1.83 | 1.91 | | 3.89 | 0.52 | 1.19 | | 4.25 | 1 | | 0.62 | 0.2 |
| Model | | -lnL* | | | | K | | AIC | | | | Delta | | | | Weight | | | CumWeight | | |
| GTR+I+G | | 136867.9 | | | | 24 | | 273783.7 | | | | 0 | | | | 0.921618 | | | 0.921618 | | |
| TVM+I+G | | 136872 | | | | 23 | | 273790.1 | | | | 6.34798 | | | | 0.038557 | | | 0.960175 | | |
| GTR+G | | 136872.1 | | | | 23 | | 273790.1 | | | | 6.39068 | | | | 0.037743 | | | 0.997918 | | |
| TVM+G | | 136876.1 | | | | 22 | | 273796.2 | | | | 12.4319 | | | | 0.001841 | | | 0.999759 | | |
| GTR+I | | 136877.2 | | | | 23 | | 273800.3 | | | | 16.60566 | | | | 0.000228 | | | 0.999987 | | |
| CDS data set | Model | | f(a) | f(c) | f(g) | | f(t) | | kappa | titv | Ra | | Rb | Rc | Rd | | Re | Rf | | pInv | gamma |
| AIC | GTR+I+G | | 0.30 | 0.17 | 0.20 | | 0.31 | | 3.87 | 1.83 | 1.91 | | 3.89 | 0.52 | 1.19 | | 4.25 | 1 | | 0.62 | 0.20 |
| Model | | -lnL* | | | | K | | AIC | | | | Delta | | | | Weight | | | CumWeight | | |
| GTR+I+G | | 136867.9 | | | | 24 | | 273783.7 | | | | 0 | | | | 0.921618 | | | 0.921618 | | |
| TVM+I+G | | 136872 | | | | 23 | | 273790.1 | | | | 6.34798 | | | | 0.038557 | | | 0.960175 | | |
| GTR+G | | 136872.1 | | | | 23 | | 273790.1 | | | | 6.39068 | | | | 0.037743 | | | 0.997918 | | |
| TVM+G | | 136876.1 | | | | 22 | | 273796.2 | | | | 12.4319 | | | | 0.001841 | | | 0.999759 | | |
| GTR+I | | 136877.2 | | | | 23 | | 273800.3 | | | | 16.60566 | | | | 0.000228 | | | 0.999987 | | |

**Table S8 Raw and trimmed read data.**

| Species | Input reads | Trimmed reads | | Total raw bases | Trimmed bases | |
| --- | --- | --- | --- | --- | --- | --- |
| *A. guttata* | 6,923,884 | 6,345,115 | 91.64% | 2,084,089,084 | 1,784,466,234 | 85.62% |
| *A. tibetana* | 6,088,966 | 5,830,039 | 95.75% | 1,832,778,766 | 1,613,977,177 | 88.06% |
| *A. euchroma* | 5,877,040 | 5,284,252 | 89.91% | 1,768,989,040 | 1,478,948,629 | 83.60% |
| *L. erythrorhizon* | 5,556,970 | 4,985,738 | 89.72% | 1,672,647,970 | 1,392,662,102 | 83.26% |

Table S9 Genome assembly information for three *Arnebia* and one *Lithospermum* chloroplast genomes.

| Species | Aligned reads (#) | Coverage (X) | Cp genome length (bp) |
| --- | --- | --- | --- |
| *A. guttata* | 325,178 | 601.79 | 150,311 |
| *A. tibetana* | 178,240 | 324.15 | 150,465 |
| *A. euchroma* | 242,602 | 447.3 | 150,250 |
| *L. erythrorhizon* | 235,626 | 433.73 | 149,316 |

Table S10 Genes in the chloroplast genomes of three *Arnebia* and one *Lithospermum* species.

| Group of genes | Name of genes |
| --- | --- |
| Photosystem I | *psaA*, *B*, *C*, *I*, *J*, *ycf3*^2^, *ycf4* |
| Photosystem II | *psbA*, *B*, *C*, *D*, *E*, *F*, *H*, *I*, *J*, *K*, *L*, *M*, *N*, *T*, *Z* |
| Cytochrome b6/f | *petA*, *B*^1^, *D*^1^, *G*, *L*, *N* |
| ATP synthase | *atpA*, *B*, *E*, *F*^1^, *H*, *I* |
| Rubisco | *rbcL* |
| NADH oxidoreductase | *ndhA*^1^, *B*^1,3^, *C*, *D*, *E*, *F*, *G*, *H*, *I*, *J*, *K* |
| Large subunit ribosomal proteins | *rpl2*^1,3^, *14*, *16*^1^, *20*, *22*, *23*^3^,*32*, *33*, *36* |
| Small subunit ribosomal proteins | *rps2*, *3*, *4*, *7*^3^, *8*, *11*, *12*^2,3,4^, *14*, *15*, *16*^1^, *18*, *19* |
| RNA polymerase | *rpoA*, *B*, *C1*^1^, *C2* |
| Unknown function protein coding gene | *ycf1*^3^, *2*^3^ |
| Other genes | *ccsA*, *cemA*, *clpP*^2^, *matK*, *infA* |
| Ribosomal RNAs | *rrn16*^3^, *23*^3^, *4.5*^3^, *5*^3^ |
| Transfer RNAs | *trnA-UGC*^1,3^, *trnC-GCA*, *trnD-GUC*, *trnE-UUC*, *trnF-GAA*, *trnfM-CAU*, *trnG-GCC*, *trnG-UCC*^1^, *trnH-GUG*, *trnI-CAU*^3^, *trnI-GAU*^1,3^, *trnK-UUU*^1^, *trnL-CAA*^3^, *trnL-UAA*^1^, *trnL-UAG*, *trnM-CAU*, *trnN-GUU*^3^, *trnP-UGG*, *trnQ-UUG*, *trnR-ACG*^3^, *trnR-UCU*, *trnS-GCU*, *trnS-GGA*, *trnS-UGA*, *trnT-GGU*, *trnT-UGU*, *trnV-GAC*^3^, *trnV-UAC*^1^, *trnW-CCA*, *trnY-GUA* |

^1^ Gene containing a single intron; ^2^ gene containing two introns; ^3^ two gene copies in IRs; ^4^ trans-splicing gene

Table S11 Genic introns in three *A. guttata* and *A. tibetana* chloroplast genomes.

| *A. guttata* | Gene | Region | Exon I | Intron I | Exon II | Intron II | Exon III |
| --- | --- | --- | --- | --- | --- | --- | --- |
| 1 | *trnk-UUU* | LSC | 37 | 2472 (2473)^1^ | 35 |  |  |
| 2 | *rps16* | LSC | 40 | 859 | 227 |  |  |
| 3 | *trnG-UCC* | LSC | 23 | 676 | 48 |  |  |
| 4 | *atpF* | LSC | 145 | 691 (690)^1^ | 410 |  |  |
| 5 | *rpoC1* | LSC | 453 | 795 | 1617 |  |  |
| 6 | *ycf3* | LSC | 124 | 730 | 230 | 772 | 153 |
| 7 | *trnL-UAA* | LSC | 35 | 483 (482)^1^ | 50 |  |  |
| 8 | *trnV-UAC* | LSC | 38 | 600 | 35 |  |  |
| 9 | *rps12* | LSC | 114 |  | 232 |  | 26 |
| 10 | *clpP* | LSC | 71 | 759 | 292 | 592 | 228 |
| 11 | *petB* | LSC | 6 | 790 | 642 |  |  |
| 12 | *petD* | LSC | 8 | 731 | 475 |  |  |
| 13 | *rpl16* | LSC | 9 | 1123 | 408 |  |  |
| 14 | *rpl2* | LSC | 391 | 649 | 434 |  |  |
| 15 | *ndhB* | IR | 777 | 670 | 756 |  |  |
| 16 | *trnI-GAU* | IR | 37 | 948 | 35 |  |  |
| 17 | *trnA-UGC* | IR | 38 | 812 | 35 |  |  |
| 18 | *ndhA* | SSC | 553 | 1023 | 539 |  |  |

^1^ Parentheses indicate *A. tibetana*

Table S12 Genic introns in three *A. euchroma* and *L. erythrorhizon* chloroplast genomes.

| *A. euchroma* | Gene | Region | Exon I | Intron I | Exon II | Intron II | Exon III |
| --- | --- | --- | --- | --- | --- | --- | --- |
| 1 | *trnk-UUU* | LSC | 37 | 2461 (2469)^1^ | 35 |  |  |
| 2 | *rps16* | LSC | 40 | 862 | 227 |  |  |
| 3 | *trnG-UCC* | LSC | 23 | 681 (676) | 48 |  |  |
| 4 | *atpF* | LSC | 145 | 691 | 410 |  |  |
| 5 | *rpoC1* | LSC | 453 | 796 (790)^1^ | 1617 |  |  |
| 6 | *ycf3* | LSC | 124 | 736 (739)^1^ | 230 | 777 (756)^1^ | 153 |
| 7 | *trnL-UAA* | LSC | 35 | 479 | 50 |  |  |
| 8 | *trnV-UAC* | LSC | 38 | 601 | 35 |  |  |
| 9 | *rps12* | LSC | 114 |  | 232 |  | 26 |
| 10 | *clpP* | LSC | 71 | 753 | 292 | 583 (594)^1^ | 228 |
| 11 | *petB* | LSC | 6 | 815 (805)^1^ | 642 |  |  |
| 12 | *petD* | LSC | 8 | 737 (730)^1^ | 475 |  |  |
| 13 | *rpl16* | LSC | 9 | 1123 (1114)^1^ | 408 |  |  |
| 14 | *rpl2* | LSC | 391 | 653 (649)^1^ | 434 |  |  |
| 15 | *ndhB* | IR | 777 | 670 | 756 |  |  |
| 16 | *trnI-GAU* | IR | 37 | 949 | 35 |  |  |
| 17 | *trnA-UGC* | IR | 38 | 812 (815)^1^ | 35 |  |  |
| 18 | *ndhA* | SSC | 553 | 1025 (1059)^1^ | 539 |  |  |

^1^ Parentheses indicate *L. erythrorhizon*
